# Comparison of protein structures by growing neighborhood alignments

**DOI:** 10.1186/1471-2105-8-77

**Published:** 2007-03-06

**Authors:** Sourangshu Bhattacharya, Chiranjib Bhattacharyya, Nagasuma R Chandra

**Affiliations:** 1 Dept. of Computer Science and Automation, Indian Institute of Science, Bangalore – 560012, India; 2 Bioinformatics Center, Indian Institute of Science, Bangalore – 560012, India

## Abstract

**Background:**

Design of protein structure comparison algorithm is an important research issue, having far reaching implications. In this article, we describe a protein structure comparison scheme, which is capable of detecting correct alignments even in difficult cases, e.g. non-topological similarities. The proposed method computes protein structure alignments by comparing, small substructures, called *neighborhoods*. Two different types of neighborhoods, sequence and structure, are defined, and two algorithms arising out of the scheme are detailed. A new method for computing equivalences having non-topological similarities from pairwise similarity score is described. A novel and fast technique for comparing sequence neighborhoods is also developed.

**Results:**

The experimental results show that the current programs show better performance on Fischer and Novotny's benchmark datasets, than state of the art programs, e.g. DALI, CE and SSM. Our programs were also found to calculate correct alignments for proteins with huge amount of indels and internal repeats. Finally, the sequence neighborhood based program was used in extensive fold and non-topological similarity detection experiments. The accuracy of the fold detection experiments with the new measure of similarity was found to be similar or better than that of the standard algorithm CE.

**Conclusion:**

A new scheme, resulting in two algorithms, have been developed, implemented and tested. The programs developed are accessible at .

## Background

It is well known that conservation of proteins at the structure level can be much higher than at the sequence level [1]. Recognizing similarities in protein structures and classifying them into folds, families, etc. is therefore an important task in biology, and is often used as a basis for designing experiments for gaining further knowledge. Unfortunately, most formulations for comparing protein structures have turned out to be NP-Hard [2]. This has led to the development of many heuristic approaches, e.g. SSAP [3], DALI [4], *C*^*α*^-match [5], LOCK [6], CE [7], SSM [8], etc.

However, it is clear from many experiments, e.g. those in [7] or results reported in the section on validation using benchmark datasets (section 0.1), that the existing algorithms produce sub-optimal results in many practical cases. For example, DALI and CE are not capable of detecting non-topological similarities. The current article reports a protein structure comparison scheme that improves upon existing algorithms in terms of quality of alignments and ability to detect non-topological similarities.

**Figure 1:**
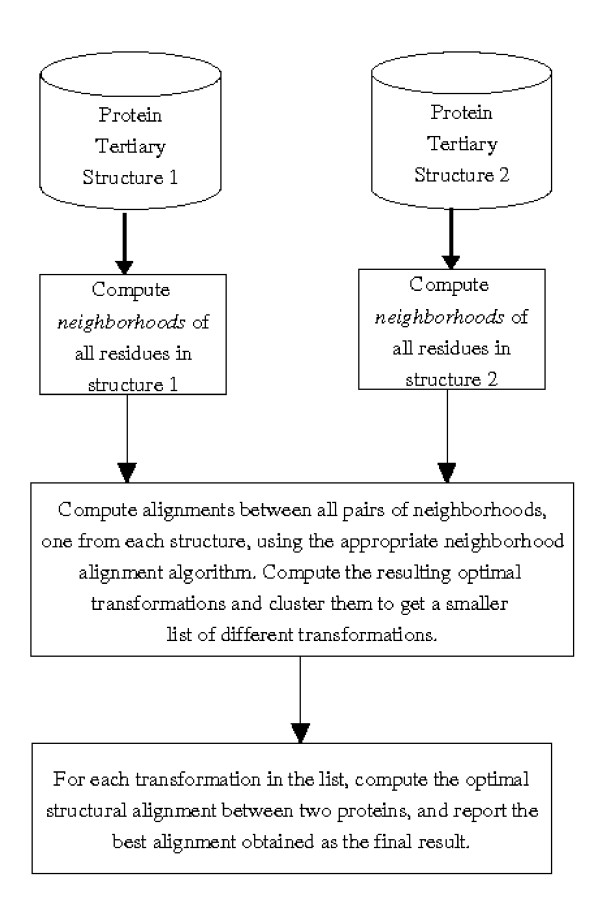
Overview of algorithms developed here.

Most protein structure comparison algorithms can be broadly classified as either distance matrix based or transformation based. This article proposes a new scheme which compares protein structures by comparing small and compact sub-structures, called *neighborhoods*. Neighborhoods spanning the entire protein are calculated for both the proteins. All pairs of neighborhoods from the two structures are aligned and resulting transformations are used to calculate the optimal alignments between the two proteins. Thus, alignments between protein structures are calculated by growing neighborhood alignments. This leads to a middle approach of comparing the neighborhoods using distance matrix based methods and calculating actual alignment using transformations obtained from neighborhood alignments (figure 1).

Following the scheme, two new algorithms based on two different kinds of neighborhoods, namely sequence and structure neighborhoods, are proposed. The structure neighborhood is a more natural concept, and gives better alignments. However, the algorithm for comparing structure neighborhoods is slow and scales exponentially with the size. The algorithm based on comparison of sequence neighborhoods is designed to be faster. For this reason, a novel and fast technique for comparing sequence neighborhoods, based on spectral graph matching [9], is developed. A more detailed description and explanation of the techniques developed is given in the methods section (section 0.4).

The techniques thus developed have been implemented, and are available for public use at [10]. The implementations were used to compare proteins from Fischer's [11] and Novotny et. al.'s [12] benchmark datasets. Results show that the current programs find similar or better alignments than DALI [4] and CE [7] in almost all cases. The current programs also show a better overall performance than SSM [8]. on the benchmark datasets. Results from comparison of proteins with large number of indels and internal repeats, show that the current algorithms detect the similarities correctly.

The sequence neighborhood based program was used in extensive fold and non-topological similarity detection experiments. For detecting fold level similarity, a new measure of significance, based on the ratio of length of alignment to the average size of the two proteins, was used. This is contrast with the normally used measures, e.g. Z-scores of some property of the alignment. The new measure is expected to work because the RMSD of the alignments have been constrained below a threshold (approx. 3 Å). The accuracy of fold detection with this new measure of similarity was found to be similar or better than that of the standard algorithm CE [7].

Finally, the current methods ability to detect non-topological similarities was tested using a circularly permuted pair of proteins. The results show that the current programs detects the full alignment, even in presence of non-topological similarities. Next section describes results from the experimental validation of the developed scheme. The scheme is described in detail, in the methods section.

## Results and Discussion

The algorithms developed in this article were implemented in C using GCC/GNU-Linux system. The programs thus developed were used to test the correctness of the concepts used in developing the algorithm on real protein structure data. This section reports the results from the experiments conducted for validating the current algorithms. Validation has been done by comparing the results from the current programs with those from the state of the art protein structure comparison programs DALI [4], CE [7], and SSM [8]. It may be noted that a recent survey [13] mentions SSM as the top performer in protein structure comparison programs.

Section 0.1, presents a systematic evaluation of the programs on two benchmark datasets containing more than 200 protein structure pairs. Section 0.2, reports tests using critical cases of proteins having multiple domains (hence large number of indels) and internally repeating subunits. In section 0.3, we explore one of the applications of protein structure comparison, i.e. the program's ability to detect proteins having the same fold as a given query protein. In section 0.4, the method's ability to detect non-topological similarities is explored.

The protein structure data was taken from PDB [14] and the SCOP [15] domains were obtained from the ASTRAL database [16]. Rasmol [17] was used to generate the molecular graphics. The programs were tested on hundreds of protein structure pairs and the optimal values of the parameters were determined so as to cater for a wide range of protein structures with varying levels of similarities. Detailed results and default parameter values are available in additional file [see Additional file 1].

### 0.1 Validation using Fischer's and Novotny's benchmark datasets

The first experiment was to validate the idea of comparing the proteins by comparing the various neighborhoods. For this, we tested the algorithms developed in this article on two benchmark datasets : Fischer [11] and Novotny et. al. [12]. Fischer's dataset was developed for testing the performance of fold recognition methods, and contains 68 pairs of protein structures having low sequence similarity. Table 1 shows the alignments generated by the two algorithms developed here and the 3 standard algorithms DALI, CE and SSM, for the 10 difficult pairs [7] of protein structures in the Fischer's dataset. It is clear that all three standard programs generate similar or worse alignments than the programs developed here on the 10 difficult protein pairs. For example, DALI generates worse alignment for the protein pairs: [PDB:2SIM] – [PDB:1NSBA], while SSM generates worse alignments for protein pairs: [PDB:1TEN] – [PDB:3HHRB], [PDB:1CID] – [PDB:2RHE] and [PDB:1BGEB] – [PDB:2GMFA]. All the other alignments generated by current programs are similar to those by their standard counterparts. This clearly demonstrates that the current programs perform competitively to the other state of the programs.

**Table 1:** 10 difficult pairs of proteins as mentioned in [7]

PDBid1(size) – PDBid2(size)	Seq Nbhd LAli/RMSD	Struct Nbhd LAli/RMSD	DALI LAli/RMSD	CE Lali/RMSD	SSM LAli/RMSD
1fxiA(96) – 1ubq(76)	54/2.18	56/2.16	60/2.6	100/3.82	60/2.86
1ten(90) – 3hhrB(185)	84/1.58	82/1.39	86/1.9	87/1.90	73/2.09
3hlaB(270) – 2rhe(114)	70/2.26	68/2.26	75/3	85/3.46	78/3.08
2azaA(129) – 1paz(120)	72/2.46	79/2.20	81/2.5	85/2.90	79/2.41
1cewI(108) – 1molA(94)	68/1.80	79/1.94	81/2.3	81/2.34	79/2.12
1cid(177) – 2rhe(114)	91/2.05	91/2.06	97/3.2	98/2.97	89/2.32
1crl(534) – 1ede(310)	160/2.50	174/2.49	211/3.5	220/3.91	188/3.81
2sim(381) – 1nsbA(390)	262/2.72	262/2.63	222/3.8	276/2.99	271/2.86
1bgeB(159) – 2gmfA(121)	85/2.48	87/2.22	94/3.3	102/4.02	44/2.49
1tie(166) – 4fgf(124)	105/2.20	106/2.27	114/3.1	115/2.86	114/2.85

Novotny et. al. [12] compared 11 fold comparison servers using pairwise comparisons for 59 proteins taken from 10 different CATH [18] topologies. Both the algorithms developed here were used to calculate the alignments for all 153 pairs of proteins in Novotny's dataset. Tables 2, 3 and 4 report a summary of comparison of results obtained from the two programs on both Fischer's and Novotny's datasets with DALI, CE and SSM, respectively. An alignment is said to be better than other if it has both higher length and lower RMSD. Two alignments are said to be level if one has both higher length and RMSD than the other. Tables 2, 3 and 4 show the number of protein pairs, for which the current method gives better/worse/level alignments than DALI, CE and SSM, respectively.

**Table 2:** Comparison of results obtained from the 2 algorithms described here with DALI

Data set/classifn.	Align. sequence nbhd. Better/Worse/Level	Align. structure nbhd. Better/Worse/Level
Fischer's	4/4/60	5/2/61

Novotny's		
1.10.164	1/0/9	2/0/8
1.10.40	11/0/10	5/0/16
1.25.30	10/0/11	5/0/16
2.30.110	0/0/6	0/0/6
2.40.100	0/0/28	0/0/28
2.100.10	5/3/7	5/0/10
3.10.70	0/0/10	0/0/10
3.40.91	0/0/6	0/0/6
3.70.10	0/0/15	2/0/13
2.40.20	0/3/18	0/0/21

**Table 3:** Comparison of results obtained from the 2 algorithms described here with CE

Data set/classifn.	Alignment of sequence nbhd. Better/Worse/Level	Alignment of structure nbhd. Better/Worse/Level
Fischer's	2/1/65	2/0/66

Novotny's		
1.10.164	0/0/10	0/0/10
1.10.40	0/0/21	0/0/21
1.25.30	1/0/20	0/0/21
2.30.110	0/0/6	0/0/6
2.40.100	6/0/22	4/0/24
2.100.10	4/0/11	4/0/11
3.10.70	1/0/9	1/0/9
3.40.91	0/0/6	0/0/6
3.70.10	0/0/15	0/1/14
2.40.20	1/1/19	0/0/21

**Table 4:** Comparison of results obtained from the 2 algorithms described here with SSM

Data set/classifin.	Alignment of sequence nbhd. Better/Worse/Level	Alignment of structure nbhd. Better/Worse/Level
Fischer's	13/10/45	23/5/40

Novotny's		
1.10.164	3/1/6	4/0/6
1.10.40	9/0/12	8/0/13
1.25.30	9/0/12	3/0/18
2.30.110	1/1/4	1/1/4
2.40.100	1/0/27	2/1/25
2.100.10	1/4/10	3/1/11
3.10.70	2/0/8	3/0/7
3.40.91	2/0/4	1/0/5
3.70.10	0/1/14	1/2/12
2.40.20	0/6/15	3/2/16

For Fischer's dataset, the sequence neighborhood based method performs similarly to DALI, whereas the structure neighborhood based method performs slightly better. However, for some CATH classes in Novotny's dataset (1.10.40, 1.25.30, 2.100.10), the current methods perform significantly better than DALI. On Fischer's dataset, the current methods consistently perform better than all the three standard programs. On Novotny's dataset, for CATH classes 1.10.164, 1.10.40 and 1.25.30, the current programs perform better than DALI and SSM, and for CATH classes 2.40.100 and 2.100.10, the current programs perform better than CE. For CATH class 3.70.10, the current programs did not give good alignments as compared to CE and SSM. However, the overall performance of the current programs is better than all the three state of the art programs. Also, it was observed (from table 1 and results provided in supplementary material) that the structure neighborhood based method performs slightly better than the sequence neighborhood based one.

### 0.2 Testing on proteins with multiple domains and internal repeats

One of the important features of proteins is the noise in the residue positions and insertions and deletions (indels) of residues. One extreme case of indels is the case when a protein has multiple domains and only one of the domains are there in the other structure. In order to test the current programs' ability of detecting such similarities, we tested them for detecting the 3 individual domains in 2 multi-domain proteins, e.g. [PDB:2HCK] and [PDB:2SRC], taken from [12]. The individual domains were obtained from the ASTRAL database. Table 5 shows the results for one of the protein (other omitted to save space). The domains were perfectly detected by both the programs developed here and all the standard programs. We also chose random fragments of length 20 residue from each of the 3 domains in [PDB:2HCK], and concatenated them to form a new structure, called "mixed" (table 5). We compared this new structure with the original protein. While both the current programs and SSM detected the match correctly, DALI and CE did not detect the correct match.

**Table 5:** Comparison of multidomain proteins with individual domains

PDBid1 – PDBid2	Seq Nbhd LAli/RMSD	Struct Nbhd LAli/RMSD	DALI LAli/RMSD	CE Lali/RMSD	SSM LAli/RMSD
2hck(438) – d2hcka1(63)	63/0.0	63/0.0	63/0.0	63/0.0	63/0.0
2hck(438) – d2hcka2(103)	103/0.0	103/0.0	103/0.0	103/0.0	103/0.0
2hck(438) – d2hcka3(272)	272/0.0	272/0.0	272/0.0	272/0.0	272/0.0

2hck(438) – mixed (60)	60/0.0	60/0.0	-/-	16/0	60/0.0

The current algorithms rely on similarity of neighborhoods to detect global similarity between proteins. However, many proteins have repeating subunits which can provide many similar looking neighborhoods. In order to ascertain whether the current programs are fooled by such internal repeats, we tested them on 6 pairs of proteins showing high degree of internal repeats. The results, reported in table 6, show that the correct alignments were detected in all the cases. Thus, we conclude that the current programs perform well, even in presence of high indels and internal repeats.

**Table 6:** Comparison of pairs of proteins with internal repeats

PDBid1 – PDBid2	Seq Nbhd LAli/RMSD	Struct Nbhd LAli/RMSD	DALI LAli/RMSD	CE LAli/RMSD	SSM LAli/RMSD
1gyhA(318) – 1tl2A(235)	155/3.00	143/2.90	196/3.6	149/3.9	192/3.99
1nscA(390) – 3sil(383)	258/2.62	257/2.67	289/3.2	272/2.9	272/2.92
1bd8(156) – 1ihbA(156)	152/1.18	153/1.22	154/1.3	154/1.3	152/1.24
1l4aA(66) – 1n7sA(63)	59/0.96	59/0.90	62/1.7	59/0.9	-/-
2pec(352) – 1bn8A(399)	269/1.62	268/1.64	287/2.5	291/2.7	258/1.59
1kapP(470) – 1sat(468)	436/1.25	438/1.24	448/1.7	446/1.5	427/1.16

### 0.3 Testing accuracy in detecting fold similarity

One of the applications of a protein structure comparison program is to compare the folds of two protein structures, or fetch all structures from a database, having the same fold as a query structure. Many programs for searching databases and comparing folds have been developed. Recently, Novotny et. al. [12] performed a comparison of fold comparison servers, and rated CE [7] as the topmost. We compared the performance of the sequence neighborhood based program (because it is faster than the structure neighborhood based one) with CE, using 5 randomly selected proteins from 5 prominent SCOP classes.

Given an alignment between 2 proteins, most programs use a statistical significance (Z-score in case of DALI and CE) to decide the level of similarity between the two proteins. In the current case, the RMSD of the alignments found by programs is upper bounded by the parameter *T *(Eqn 1). For *T *= 5 (default) the RMSD is found to be less than 3 Å. Thus, the free parameter is the length of the alignment. We used the percentage of residues aligned as a fraction of the average number of residues to decide the level of similarity. Thus for each alignment, we calculate percent aligned as: *percalign *= lenlav
 MathType@MTEF@5@5@+=feaafiart1ev1aaatCvAUfKttLearuWrP9MDH5MBPbIqV92AaeXatLxBI9gBaebbnrfifHhDYfgasaacH8akY=wiFfYdH8Gipec8Eeeu0xXdbba9frFj0=OqFfea0dXdd9vqai=hGuQ8kuc9pgc9s8qqaq=dirpe0xb9q8qiLsFr0=vr0=vr0dc8meaabaqaciaacaGaaeqabaqabeGadaaakeaadaWcaaqaaiabdYgaSjabdwgaLjabd6gaUbqaaiabdYgaSnaaBaaaleaacqWGHbqycqWG2bGDaeqaaaaaaaa@3522@ × 100, where *l*_*av *_= (*m *+ *n*)/2.

Table 7 shows the results of a database search on the 40% non-redundant ASTRAL database using the 5 query proteins. The output from sequence neighborhood based program was filtered using 2 percentage aligned cutoff values, e.g. 50% and 45%. For CE, the prescribed cutoff of 4.0 on Z-score value was used. The true and false positives, and the actual number of entries in the database are reported. For d101m__ and d1htia the number of false positives given by CE are much higher than the current program, whereas for d1jzba_ and d2pela_ fewer true examples are detected by CE. On the whole, the current program performs at par with CE without even invoking any statistical theory.

**Table 7:** Detection of folds using sequence neighborhood alignment method

SCOPid (tot. num.)	Method	cutoff (%/Z)	True +ve	False +ve
d101m__(37)	seq nbhd	50%	34	9
	seq nbhd	45%	35	38
	CE	4.0	35	95
d1htia_(253)	seq nbhd	50%	190	4
	seq nbhd	45%	231	13
	CE	4.0	233	224
d1jzba_(119)	seq nbhd	50%	28	56
	seq nbhd	45%	48	172
	CE	4.0	2	0
d2pela_ (48)	seq nbhd	50%	41	9
	seq nbhd	45%	45	21
	CE	4.0	36	8
d7rsa__(4)	seq nbhd	50%	4	0
	seq nbhd	45%	4	13
	CE	4.0	4	0

### 0.4 Determination of non-topological similarities

**Figure 2:**
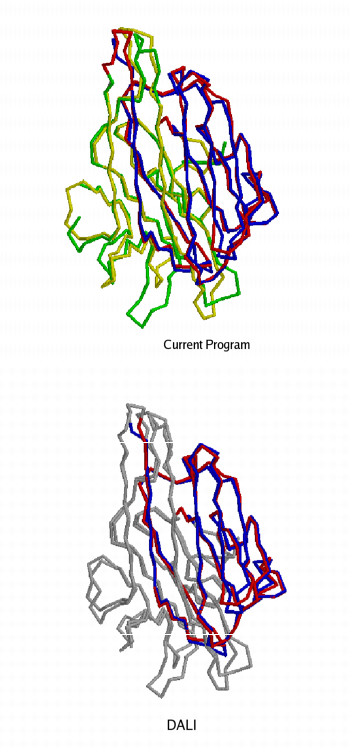
2pelA – 5cnaA alignments generated by the current program and DALI.

Non-topological similarities between proteins is an important phenomenon, from both scientific and practical applications points of view [19,20]. In this section, we explore the capability of the current programs to detect non-topological similarities. The current programs were tested with the well known circularly permuted pair of proteins [PDB:2PEL] – [PDB:5CNA], showing very high structural similarity. Figure 2 reports the alignments and superpositions from the current programs (in this case both the current programs report the same alignment) and DALI. The current programs give a 219 residue alignment with RMSD 1.3 Å. whereas DALI reports 117 residue alignment with RMSD 1.3 Å. It is clear from figure 2 that DALI detects only a portion of the actual similarity. CE detects a 116 residue alignment with 1.2 ÅRMSD and SSM detects a 116 residue alignment with 1.23 ÅRMSD. Thus it is clear that none on the three programs used to benchmark the current programs detect full alignment in presence of non-topological similarities.

In order to find out more naturally occurring circularly permuted pairs of proteins, we compared all possible pairs of proteins, showing less than 40% sequence similarity, from the SCOP fold b.29. There are 48 domains in the b.29 fold of 40% non-redudant ASTRAL dataset. Out of all the 1128 pairs, 107 pairs showed circular permutations. Table 8 shows 5 circularly permutated pairs of proteins having low structural similarity. In 4 of the 5 cases, the current program detects a better alignment than DALI, and in one case DALI doesn't detect any significant alignment. A detailed study of these circular permutations will be reported elsewhere. Thus, it is clear that the current programs can be useful as a tool for detecting circular permutations occuring in nature.

**Table 8:** 5 examples of circular permutations detected with low structural similarity

SCOPid1 (size)	SCOPid2 (size)	seq nbhd LAli/RMSD	DALI LAli/RMSD
d1t6gc_(182)	d1okqa2(185)	92/2.77	-/-
d1olra_(226)	d1w0pa2(197)	107/2.72	101/3.8
d1dypa_(266)	d1nls__(238)	129/2.39	83/3.0
d1s2ba_(199)	d1epwa1(218)	107/2.87	117/3.9
d1wd3a1(319)	d1mvea_(238)	143/2.75	133/3.7

## Conclusion

Biology, in its present practice, is effectively a relational science. Decisions made with one system being heavily influenced by the knowledge obtained from other systems. It is quite understandable therefore, why recognizing similarities and deriving relationships are crucial for all further knowledge. In this context, protein structure comparison is an important yet complex problem.

The current article proposes a new scheme for comparing protein structures, relying on efficient techniques available for comparing smaller sub-structures (called neighborhoods), having equal number of residues. This reduces the problem of comparing protein structures to that of searching over at most *O*(*n*^2^) transformations, under the assumption that at least one of the neighborhoods from the first protein has a match in the second protein.

The scheme leads to two specific algorithms, by using two types of neighborhood definitions. The structure neighborhood is the most intuitive, and is seen (section 0.1) to give better alignments in many cases. However, the algorithm based on structure neighborhoods is seen to be approximately 2–4 times slower than the sequence neighborhood based algorithm. Also, with sequence neighborhood larger neighborhood sizes could be explored (17 as opposed to 6 for structure neighborhoods). This is possible due to a novel technique developed (in section 0.10) for comparing sequence neighborhoods. A web interface to the programs developed is available at [10].

The algorithms reported here overcome some of the drawbacks seen with other approaches, such as the detection of non-topological similarities. Also, with the current approach, the alignment quality has been found to be superior to the state of the art programs, e.g. DALI, CE and SSM, in a number of cases. So, as demonstrated in the results, the proposed algorithms will aid in identifying structural similarities that may have been missed out with other approaches.

The current article also simplifies detection of folds by using ratio of length of alignment and the average length of the two sequences, as opposed to Z-scores of some quantity used by other programs. This is done by constraining the RMSD to be below a cutoff. The program uses simple rules based on RMSD and length of alignment to hit upon the best alignment. Such simplification makes the hits returned by the program easier to interpret (as opposed to complex statistical significance measures). Even with the simplified measures, the accuracy of fold detection for the current program was found to be better than CE [7] in some cases.

## Methods

A protein structure is described by the 3D coordinates of all non-hydrogen atoms present in the protein. However, following the common practice, we will use the coordinates of the *C*^*α *^atom of each residue to describe each protein structure. So, a protein structure *A*, having *m *residues, will be described as *A *= {**x**_1_, **x**_2_,...,**x**_*m*_}, **x**_*i *_∈ ℝ^3^, 1 ≤ *i *≤ *m*, where each point **x**_*i *_represents the position of one residue. The sequence ordering of the polypeptide is given by the indices of the points. Similarly, the second protein *B *is represented as *B *= {**y**_1_,...,**y**_*n*_}, **y**_*j*_, ∈ ℝ^3^, 1 ≤ *j *≤ *n*.

A *structural alignment *between two protein structures *A *and *B *is given by a set of *equivalences *(1 - 1 correspondences) between the residues of the 2 proteins. So, a structural alignment Φ between structures *A *and *B*, of length *L*, is denoted as:

Φ (*A*, *B*) = {(*i*_*l*_, *j*_*l*_)|1 ≤ *l *≤ *L*, 1 ≤ *i*_*l *_≤ *m*, 1 ≤ *j*_*l *_≤ *n*, and *i*_*l *_= *i*_*k *_or *j*_*l *_= *j*_*k *_iff *l *= *k*}. In the alignment the ilth
 MathType@MTEF@5@5@+=feaafiart1ev1aaatCvAUfKttLearuWrP9MDH5MBPbIqV92AaeXatLxBI9gBaebbnrfifHhDYfgasaacH8akY=wiFfYdH8Gipec8Eeeu0xXdbba9frFj0=OqFfea0dXdd9vqai=hGuQ8kuc9pgc9s8qqaq=dirpe0xb9q8qiLsFr0=vr0=vr0dc8meaabaqaciaacaGaaeqabaqabeGadaaakeaacqWGPbqAdaqhaaWcbaGaemiBaWgabaGaemiDaqNaemiAaGgaaaaa@325F@ residue of protein *A *is said to be matched or equivalenced with the jlth
 MathType@MTEF@5@5@+=feaafiart1ev1aaatCvAUfKttLearuWrP9MDH5MBPbIqV92AaeXatLxBI9gBaebbnrfifHhDYfgasaacH8akY=wiFfYdH8Gipec8Eeeu0xXdbba9frFj0=OqFfea0dXdd9vqai=hGuQ8kuc9pgc9s8qqaq=dirpe0xb9q8qiLsFr0=vr0=vr0dc8meaabaqaciaacaGaaeqabaqabeGadaaakeaacqWGQbGAdaqhaaWcbaGaemiBaWgabaGaemiDaqNaemiAaGgaaaaa@3261@ residue of protein *B*. In this definition, there is no notion of maintaining the topology. However, some programs find only topological similarities, and thus require the condition *i*_*l *_<*i*_*k *_iff *j*_*l *_<*j*_*k *_to be satisfied. A *graph G *with vertex set *V *and edge set *E *⊆ *V *× *V *is denoted as *G *= (*V*, *E*).

The *score *of a structural alignment is a measure of its suitability for the current purpose. Many scoring functions have been developed and used by different programs. Also, many programs do not specify a global scoring function, but optimize a local property at each stage. In the next section, we analyze the algorithms and scoring functions of different programs for comparing protein structures, and summarize their desired characteristics.

### 0.5 Analysis of different algorithms

Methods for comparing protein structures can be broadly divided into two types : ones that calculate the optimal rigid transformation of one structure on the other (e.g. LOCK), and ones that do not explicitly calculate the transformation. Distance matrix based methods (e.g. DALI and CE) are popular among the second type of methods.

The problem with methods based on distance matrices is that searching over all possible alignments blows up exponentially with the number of residues. DALI uses a Monte-Carlo optimization technique to search the space of all alignments in a randomized way. CE reduces the search space by using sequence constraints and heuristic cutoffs on extension of alignments.

Methods based on transformation calculation search over the space of all transformations, which is a continuous space. However, due to the multi modal nature of the scoring function over the space of transformations, local search based methods risk converging to a locally optimum solution. Kolodny and Linial [21] suggest carefully discretizing the space of all transformations, based on a given approximation parameter to the objective function, arriving at a polynomial time approximation algorithm for comparing protein structures. Unfortunately, the time complexity of the algorithm is very high for practical applications. *C*^*α*^-match also uses discretization of the search space using the geometric hashing technique. It has been observed that (see section 0.4) the conformation search based algorithms are better at finding non-topological similarities than their distance matrix based counterparts. This is typically due to constraining of search space to curb the exponential blowup. However, distance matrix based algorithms have the potential to solve the problem to its global optimum.

In this article, we take an intermediate approach using distance matrix based methods to align sub-structures, called neighborhoods, and arrive at the optimal transformation starting from the transformations calculated using sub-structure alignments. The alignments between the neighborhoods provide a discrete set of transformations to search from. To this end, we define the notion of neighborhoods and motivate the comparison of neighborhoods for comparing protein structures, in the next section.

### 0.6 Neighborhoods and their comparison

It is well known that atoms close to a particular atom, in the three dimensional structure of a protein, have more influence on the position of the current atom than those that are farther apart. This fact has been utilized by the DALI score function to weight the errors corresponding to distances with an inverse exponential function of the average distance. This fact has also been utilized by *C*^*α*^-match in introducing heuristic cutoffs on the distance between the residues forming reference frames, and those being considered for voting.

We use this fact more strongly to define the notion of neighborhood of a residue. The *k-structure neighborhood *of a residue of a protein is defined as the set of *k *residues nearest to the given residue in 3D. Thus, the neighborhood NstrA
 MathType@MTEF@5@5@+=feaafiart1ev1aaatCvAUfKttLearuWrP9MDH5MBPbIqV92AaeXatLxBI9gBaebbnrfifHhDYfgasaacH8akY=wiFfYdH8Gipec8Eeeu0xXdbba9frFj0=OqFfea0dXdd9vqai=hGuQ8kuc9pgc9s8qqaq=dirpe0xb9q8qiLsFr0=vr0=vr0dc8meaabaqaciaacaGaaeqabaqabeGadaaakeaacqWGobGtdaqhaaWcbaGaem4CamNaemiDaqNaemOCaihabaGaemyqaeeaaaaa@3356@(*i*) of the *i*^*th *^residue of *A *is characterized by NstrA
 MathType@MTEF@5@5@+=feaafiart1ev1aaatCvAUfKttLearuWrP9MDH5MBPbIqV92AaeXatLxBI9gBaebbnrfifHhDYfgasaacH8akY=wiFfYdH8Gipec8Eeeu0xXdbba9frFj0=OqFfea0dXdd9vqai=hGuQ8kuc9pgc9s8qqaq=dirpe0xb9q8qiLsFr0=vr0=vr0dc8meaabaqaciaacaGaaeqabaqabeGadaaakeaacqWGobGtdaqhaaWcbaGaem4CamNaemiDaqNaemOCaihabaGaemyqaeeaaaaa@3356@(*i*) = {**x **∈ *A*|**x' **∉ NstrA
 MathType@MTEF@5@5@+=feaafiart1ev1aaatCvAUfKttLearuWrP9MDH5MBPbIqV92AaeXatLxBI9gBaebbnrfifHhDYfgasaacH8akY=wiFfYdH8Gipec8Eeeu0xXdbba9frFj0=OqFfea0dXdd9vqai=hGuQ8kuc9pgc9s8qqaq=dirpe0xb9q8qiLsFr0=vr0=vr0dc8meaabaqaciaacaGaaeqabaqabeGadaaakeaacqWGobGtdaqhaaWcbaGaem4CamNaemiDaqNaemOCaihabaGaemyqaeeaaaaa@3356@(*i*) ⇒ ||**x' **- **x**_*i*_|| ≤ ||**x' **- **x**_*i*_|| and |NstrA
 MathType@MTEF@5@5@+=feaafiart1ev1aaatCvAUfKttLearuWrP9MDH5MBPbIqV92AaeXatLxBI9gBaebbnrfifHhDYfgasaacH8akY=wiFfYdH8Gipec8Eeeu0xXdbba9frFj0=OqFfea0dXdd9vqai=hGuQ8kuc9pgc9s8qqaq=dirpe0xb9q8qiLsFr0=vr0=vr0dc8meaabaqaciaacaGaaeqabaqabeGadaaakeaacqWGobGtdaqhaaWcbaGaem4CamNaemiDaqNaemOCaihabaGaemyqaeeaaaaa@3356@(*i*)| = *k*}. Note that **x**_*j *_∈ NstrA
 MathType@MTEF@5@5@+=feaafiart1ev1aaatCvAUfKttLearuWrP9MDH5MBPbIqV92AaeXatLxBI9gBaebbnrfifHhDYfgasaacH8akY=wiFfYdH8Gipec8Eeeu0xXdbba9frFj0=OqFfea0dXdd9vqai=hGuQ8kuc9pgc9s8qqaq=dirpe0xb9q8qiLsFr0=vr0=vr0dc8meaabaqaciaacaGaaeqabaqabeGadaaakeaacqWGobGtdaqhaaWcbaGaem4CamNaemiDaqNaemOCaihabaGaemyqaeeaaaaa@3356@(*i*) might not be connected in sequence.

Another notion of neighborhood which maintains sequence connectivity is the *sequence neighborhood*, defined as a fragment of sequence of a given length. Thus, a *k*-sequence neighborhood, NseqA
 MathType@MTEF@5@5@+=feaafiart1ev1aaatCvAUfKttLearuWrP9MDH5MBPbIqV92AaeXatLxBI9gBaebbnrfifHhDYfgasaacH8akY=wiFfYdH8Gipec8Eeeu0xXdbba9frFj0=OqFfea0dXdd9vqai=hGuQ8kuc9pgc9s8qqaq=dirpe0xb9q8qiLsFr0=vr0=vr0dc8meaabaqaciaacaGaaeqabaqabeGadaaakeaacqWGobGtdaqhaaWcbaGaem4CamNaemyzauMaemyCaehabaGaemyqaeeaaaaa@3336@(*i*) starting from residue *i *of structure *A *is defined as NseqA
 MathType@MTEF@5@5@+=feaafiart1ev1aaatCvAUfKttLearuWrP9MDH5MBPbIqV92AaeXatLxBI9gBaebbnrfifHhDYfgasaacH8akY=wiFfYdH8Gipec8Eeeu0xXdbba9frFj0=OqFfea0dXdd9vqai=hGuQ8kuc9pgc9s8qqaq=dirpe0xb9q8qiLsFr0=vr0=vr0dc8meaabaqaciaacaGaaeqabaqabeGadaaakeaacqWGobGtdaqhaaWcbaGaem4CamNaemyzauMaemyCaehabaGaemyqaeeaaaaa@3336@(*i*) = {**x**_*i*_,...,**x**_*i*+*k*-1_}. The main reason for defining sequence neighborhoods is that (see section 0.10), it is easier to match sets of points with sequence order than points without any order. Also, while matching two structures, one of the structures can be tiled along the sequence using sequence neighborhoods. So, instead of *n *- *k *+ 1 (as in the case when considering all possible sequence neighborhoods), ⌈(*n/k*)⌉ different neighborhoods are to matched. Thus, sequence neighborhoods can be used to reduce the running time of the algorithm, and also allow larger neighborhood sizes.

In the next section, we describe the algorithm for matching structures based on alignments of neighborhoods. The algorithms for aligning neighborhoods will be described in subsequent sections. An important point to note here is that even for the sequence neighborhoods, we calculate the structural alignments. Thus, there can be non-topological similarities in the alignments of neighborhoods.

### 0.7 Alignment of protein structures using neighborhood alignments

The central idea behind computation of optimal structural alignment from neighborhood alignments is that the optimal structural alignment will have at least one of the neighborhoods of residues aligned almost fully and optimally. In other words, the optimal superposition between two protein structures will contain an almost full (with very few indels) and optimal superposition of two neighborhoods. This assumption is valid under various circumstances, e.g. for proteins having functional similarity, the sites responsible for functions are conserved. Also, proteins having similar folds will have a highly conserved core or conserved regions of secondary structures.

Utilizing these ideas, we propose the following scheme for comparing two protein structures by systematically comparing neighborhoods.

1. For each of the two proteins, the neighborhoods (either structure or sequence) are selected to span the entire protein.

2. All pairs of neighborhoods, with one neighborhood from each of the two proteins, are aligned optimally using the appropriate neighborhood alignment algorithm.

3. The aligned neighborhoods are then optimally superposed, and the resulting transformations of first protein on the second are stored in a list.

4. Using the transformations calculated above, all residues of the first protein are transformed.

5. For each of the transformations, a pairwise similarity score is calculated between the residues of the two proteins using the transformed coordinates of the first protein and the original coordinates of the second.

6. The equivalences corresponding to the similarity scores are calculated using *greedy fragment pair search*. The best set of equivalences (structural alignment) thus calculated is reported.

In step 1, while choosing structure neighborhoods, one centered at every atom is chosen making *m *and *n *neighborhoods respectively. While choosing sequence neighborhoods, we tile one of the structures with the chosen neighborhoods, i.e. choose neighborhoods starting at positions (1 + *ik*) for *i *= 1... ⌊*n/k*⌋, and the last one starting from *n *- *k *+ 1 to include the remaining residues if any. On the second structure, all the neighborhoods starting from 1...(*n *- *k *+ 1) are chosen.

For step 2, the algorithm for matching structure and sequence neighborhoods are described in sections 0.9 and 0.10, respectively. While considering neighborhood alignments for calculating transformations (step 3), only the alignments having length (number of aligned residues) greater than a cutoff (*LenCutoff*) is considered. This is because the neighborhood comparison algorithms will return some alignment, even between dissimilar neighborhoods. Moreover, at least 3 aligned residue pairs are required to calculate transformations in 3D. Also, note that irrespective of the neighborhood type, neighborhood alignments match the 3D structure of the neighborhood. Thus, even in case of sequence neighborhood, the final alignment is not dependent of the sequence similarity.

The transformations in step 3 are calculated using the method described in [22]. The method uses unit quaternions (4 component vectors with norm 1) to describe rotations and a vector in 3D for describing the translation of the origin. Thus, every transformation T
 MathType@MTEF@5@5@+=feaafiart1ev1aaatCvAUfKttLearuWrP9MDH5MBPbIqV92AaeXatLxBI9gBamrtHrhAL1wy0L2yHvtyaeHbnfgDOvwBHrxAJfwnaebbnrfifHhDYfgasaacH8akY=wiFfYdH8Gipec8Eeeu0xXdbba9frFj0=OqFfea0dXdd9vqai=hGuQ8kuc9pgc9s8qqaq=dirpe0xb9q8qiLsFr0=vr0=vr0dc8meaabaqaciaacaGaaeqabaWaaeGaeaaakeaaimaacqWFtepvaaa@3847@ is a 7 component vector, T
 MathType@MTEF@5@5@+=feaafiart1ev1aaatCvAUfKttLearuWrP9MDH5MBPbIqV92AaeXatLxBI9gBamrtHrhAL1wy0L2yHvtyaeHbnfgDOvwBHrxAJfwnaebbnrfifHhDYfgasaacH8akY=wiFfYdH8Gipec8Eeeu0xXdbba9frFj0=OqFfea0dXdd9vqai=hGuQ8kuc9pgc9s8qqaq=dirpe0xb9q8qiLsFr0=vr0=vr0dc8meaabaqaciaacaGaaeqabaWaaeGaeaaakeaaimaacqWFtepvaaa@3847@ ∈ ℝ^7^.

Many transformations calculated in step 3, will be the same except for small numerical differences. In order to avoid processing a transformation multiple times, the transformations are clustered, and one transformation from each cluster is considered for further processing. Since the transforming the points involve multiplication by a polynomial of degree two in the quaternion components (see [22]), small changes in the component values (maintaining unit norm property) will produce small changes in the transformed coordinates. This property is used to cluster the transformations based on 2-norm. For clustering, a random order is chosen for the transformations and the first transformation is chosen as the center of a cluster. The subsequent transformations are put assigned clusters using the scheme: a transformation T
 MathType@MTEF@5@5@+=feaafiart1ev1aaatCvAUfKttLearuWrP9MDH5MBPbIqV92AaeXatLxBI9gBamrtHrhAL1wy0L2yHvtyaeHbnfgDOvwBHrxAJfwnaebbnrfifHhDYfgasaacH8akY=wiFfYdH8Gipec8Eeeu0xXdbba9frFj0=OqFfea0dXdd9vqai=hGuQ8kuc9pgc9s8qqaq=dirpe0xb9q8qiLsFr0=vr0=vr0dc8meaabaqaciaacaGaaeqabaWaaeGaeaaakeaaimaacqWFtepvaaa@3847@', belongs to a cluster *i*, if ||T
 MathType@MTEF@5@5@+=feaafiart1ev1aaatCvAUfKttLearuWrP9MDH5MBPbIqV92AaeXatLxBI9gBamrtHrhAL1wy0L2yHvtyaeHbnfgDOvwBHrxAJfwnaebbnrfifHhDYfgasaacH8akY=wiFfYdH8Gipec8Eeeu0xXdbba9frFj0=OqFfea0dXdd9vqai=hGuQ8kuc9pgc9s8qqaq=dirpe0xb9q8qiLsFr0=vr0=vr0dc8meaabaqaciaacaGaaeqabaWaaeGaeaaakeaaimaacqWFtepvaaa@3847@' - T
 MathType@MTEF@5@5@+=feaafiart1ev1aaatCvAUfKttLearuWrP9MDH5MBPbIqV92AaeXatLxBI9gBamrtHrhAL1wy0L2yHvtyaeHbnfgDOvwBHrxAJfwnaebbnrfifHhDYfgasaacH8akY=wiFfYdH8Gipec8Eeeu0xXdbba9frFj0=OqFfea0dXdd9vqai=hGuQ8kuc9pgc9s8qqaq=dirpe0xb9q8qiLsFr0=vr0=vr0dc8meaabaqaciaacaGaaeqabaWaaeGaeaaakeaaimaacqWFtepvaaa@3847@_*i*_|| ≤ *ClustThreshold*, T
 MathType@MTEF@5@5@+=feaafiart1ev1aaatCvAUfKttLearuWrP9MDH5MBPbIqV92AaeXatLxBI9gBamrtHrhAL1wy0L2yHvtyaeHbnfgDOvwBHrxAJfwnaebbnrfifHhDYfgasaacH8akY=wiFfYdH8Gipec8Eeeu0xXdbba9frFj0=OqFfea0dXdd9vqai=hGuQ8kuc9pgc9s8qqaq=dirpe0xb9q8qiLsFr0=vr0=vr0dc8meaabaqaciaacaGaaeqabaWaaeGaeaaakeaaimaacqWFtepvaaa@3847@_*i *_being cluster center of the *i*^*th *^cluster. Otherwise, it forms a new cluster center. This scheme avoids processing similar transformations multiple times. Steps 4–6 are essentially growing the neighborhood alignments corresponding to the transformations obtained above to get the full alignments. This is done by first transforming the residues of one structure onto other so that their positions can be compared directly (step 4), followed by calculation the similarity scores between residues of the two structures (step 5). The similarity score is given by the equation:

*S*(*i*, *j*) = *T *- ||T
 MathType@MTEF@5@5@+=feaafiart1ev1aaatCvAUfKttLearuWrP9MDH5MBPbIqV92AaeXatLxBI9gBamrtHrhAL1wy0L2yHvtyaeHbnfgDOvwBHrxAJfwnaebbnrfifHhDYfgasaacH8akY=wiFfYdH8Gipec8Eeeu0xXdbba9frFj0=OqFfea0dXdd9vqai=hGuQ8kuc9pgc9s8qqaq=dirpe0xb9q8qiLsFr0=vr0=vr0dc8meaabaqaciaacaGaaeqabaWaaeGaeaaakeaaimaacqWFtepvaaa@3847@ (**x**_*i*_) - **y**_*j*_||, 1 ≤ *i *≤ *m*, 1 ≤ *j *≤ *n *    (1)

where, *T *is the tolerance in distance between the two superposed residues, and T
 MathType@MTEF@5@5@+=feaafiart1ev1aaatCvAUfKttLearuWrP9MDH5MBPbIqV92AaeXatLxBI9gBamrtHrhAL1wy0L2yHvtyaeHbnfgDOvwBHrxAJfwnaebbnrfifHhDYfgasaacH8akY=wiFfYdH8Gipec8Eeeu0xXdbba9frFj0=OqFfea0dXdd9vqai=hGuQ8kuc9pgc9s8qqaq=dirpe0xb9q8qiLsFr0=vr0=vr0dc8meaabaqaciaacaGaaeqabaWaaeGaeaaakeaaimaacqWFtepvaaa@3847@ is the transformation being considered. Finally, the equivalences are calculated based on the above similarity score using the *greedy fragment pair search *technique described in section 0.8. Given the equivalences, the optimal superposition and hence RMSD are computed using the method described in [22].

For each transformation in the clustered list, the structural alignment between two structures is calculated as described above. The best transformation (and hence alignment) is decided based on RMSD and length of alignment, using the following rules:

For two alignments *Al*_1 _and *Al*_2_:

• If *RMSD*_1 _≤ *RMSD*_2 _and *len*_1 _≥ *len*_2 _then *Al*_1 _is better than *A1*_2 _and vice versa.

• If *RMSD*_1 _≤ *RMSD*_2 _and *len*_1 _≥ *len*_2 _then, if (len2−len1)(RMSD2−RMSD1)
 MathType@MTEF@5@5@+=feaafiart1ev1aaatCvAUfKttLearuWrP9MDH5MBPbIqV92AaeXatLxBI9gBaebbnrfifHhDYfgasaacH8akY=wiFfYdH8Gipec8Eeeu0xXdbba9frFj0=OqFfea0dXdd9vqai=hGuQ8kuc9pgc9s8qqaq=dirpe0xb9q8qiLsFr0=vr0=vr0dc8meaabaqaciaacaGaaeqabaqabeGadaaakeaadaWcaaqaaiabcIcaOiabdYgaSjabdwgaLjabd6gaUnaaBaaaleaacqaIYaGmaeqaaOGaeyOeI0IaemiBaWMaemyzauMaemOBa42aaSbaaSqaaiabigdaXaqabaGccqGGPaqkaeaacqGGOaakcqWGsbGucqWGnbqtcqWGtbWucqWGebardaWgaaWcbaGaeGOmaidabeaakiabgkHiTiabdkfasjabd2eanjabdofatjabdseaenaaBaaaleaacqaIXaqmaeqaaOGaeiykaKcaaaaa@47E8@ > *LRcutoff *then *Al*_2 _is better else *Al*_1 _is better; and vice versa.

The alignment given by this algorithm will have RMSD lower than the value of parameter *T *(eqn. 1) because, residue pairs separated by a distance more than *T *will have a negative similarity score, and thus will not be taken up as aligned residues. So, the parameter *T *can be used to control the RMSD of the match, depending on the application. The above algorithm computes *mn *neighborhood alignments for computing transformations. However, the alignment length cutoff used after step 2, and clustering of transformations in step 3 reduce the effectie number of transformations to be processed to *O*(*n*). The effective complexity of processing each transformation is *O*(*n*^2^) (see section 0.8). Thus the effective overall time complexity is *O*(*n*^3^).

Two key components of the above technique are the algorithm for calculating alignment given similarity scores between the residues of the two proteins, and the algorithms for calculating neighborhood alignments. The former is described in next section and the later in the subsequent two sections.

### 0.8 Greedy fragment pair search

In this section, we propose an algorithm for calculating the equivalences between residues of two proteins *A *and *B *given a similarity score *S*(*i*, *j*), 1 ≤ *i *≤ *m*, 1 ≤ *j *≤ *n*. The algorithm is inspired from the local alignment algorithm [23], which detects the highest scoring pair of fragments between two sequences. However, only similarities that follow the sequence order are detected. We modify the algorithm to detect multiple fragments which may not follow sequence ordering.

Ideally, given an alignment Φ(*A*, *B*), one would want to maximize the score ∑l=1LS(il,jl)
 MathType@MTEF@5@5@+=feaafiart1ev1aaatCvAUfKttLearuWrP9MDH5MBPbIqV92AaeXatLxBI9gBaebbnrfifHhDYfgasaacH8akY=wiFfYdH8Gipec8Eeeu0xXdbba9frFj0=OqFfea0dXdd9vqai=hGuQ8kuc9pgc9s8qqaq=dirpe0xb9q8qiLsFr0=vr0=vr0dc8meaabaqaciaacaGaaeqabaqabeGadaaakeaadaaeWaqaaiabdofatjabcIcaOiabdMgaPnaaBaaaleaacqWGSbaBaeqaaOGaeiilaWIaemOAaO2aaSbaaSqaaiabdYgaSbqabaGccqGGPaqkaSqaaiabdYgaSjabg2da9iabigdaXaqaaiabdYeambqdcqGHris5aaaa@3CCD@. However, this problem (the assignment problem) is difficult and has a very slow solution. Moreover, this does not use the information available in the sequence ordering of the protein. We propose to detect high scoring fragment pairs in a *greedy *way (i.e. the pick the best first, followed by second best, and so on). For this, the local alignment matrix is calculated as:

Li,j={0,if i=0or j=0max⁡{Li−1,j−1+s(i,j)Li−1,j−gLi,j−1−g0},otherwise     (2)
 MathType@MTEF@5@5@+=feaafiart1ev1aaatCvAUfKttLearuWrP9MDH5MBPbIqV92AaeXatLxBI9gBaebbnrfifHhDYfgasaacH8akY=wiFfYdH8Gipec8Eeeu0xXdbba9frFj0=OqFfea0dXdd9vqai=hGuQ8kuc9pgc9s8qqaq=dirpe0xb9q8qiLsFr0=vr0=vr0dc8meaabaqaciaacaGaaeqabaqabeGadaaakeaacqWGmbatdaWgaaWcbaGaemyAaKMaeiilaWIaemOAaOgabeaakiabg2da9maaceqabaqbaeqabiGaaaqaaiabicdaWaqaaiabcYcaSuaabaqaceaaaeaacqqGPbqAcqqGMbGzcqqGGaaicqWGPbqAcqGH9aqpcqaIWaamaeaacqqGVbWBcqqGYbGCcqqGGaaicqWGQbGAcqGH9aqpcqaIWaamaaaabaGagiyBa0MaeiyyaeMaeiiEaG3aaiWabeaafaqaaeabbaaaaeaacqWGmbatdaWgaaWcbaGaemyAaKMaeyOeI0IaeGymaeJaeiilaWIaemOAaOMaeyOeI0IaeGymaedabeaakiabgUcaRiabdohaZjabcIcaOiabdMgaPjabcYcaSiabdQgaQjabcMcaPaqaaiabdYeamnaaBaaaleaacqWGPbqAcqGHsislcqaIXaqmcqGGSaalcqWGQbGAcqGHsislcqWGNbWzaeqaaaGcbaGaemitaW0aaSbaaSqaaiabdMgaPjabcYcaSiabdQgaQjabgkHiTiabigdaXaqabaGccqGHsislcqWGNbWzaeaacqaIWaamaaaacaGL7bGaayzFaaaabaGaeiilaWIaee4Ba8MaeeiDaqNaeeiAaGMaeeyzauMaeeOCaiNaee4DaCNaeeyAaKMaee4CamNaeeyzaugaaaGaay5EaaGaaCzcaiaaxMaadaqadaqaaiabikdaYaGaayjkaiaawMcaaaaa@7E1C@

The highest entry in the matrix corresponds to the highest scoring fragment pair, and the corresponding fragment pair can be detected by tracing back from the highest scoring entry. However, the matrix also contains scores for other high scoring fragment pairs. The second highest scoring fragment pair is detected after deleting rows and columns corresponding to the residues in the highest scoring fragment pair. This iterative procedure continued till there are no positive scoring entries in the matrix or all the rows and columns have been deleted. The steps are given in Algorithm 1. Generation of the local alignment matrix takes *O*(*n*^2^) time. Each search through the matrix for highest scoring entry also takes *O*(*n*^2^) time, and the traceback takes *O*(*n*) time, thereby giving an *O*(*n*^3^) bound on complexity. However, since in most cases only a small number of large fragment pairs will be picked up, the effective time complexity is *O*(*n*^2^).

### 0.9 Aligning structure neighborhoods

A *k*-structure neighborhood of a residue consists of the *k *residues nearest to the current residue. So, there may not be any sequence connection between these residues. Thus, we look at the structure neighborhood

**Algorithm 1 **Greedy Fragment Pair Search

1: *Alignment *← *φ*.

2: Compute *highest *= *max*_*i*, *j *_*L*_*i*, *j*_.

3: Compute (*u*, *v*) = *arg *max_*i*, *j *_*L*_*i*, *j*_.

4: while *highest *> 0 do

5:    *Alignment *← *Alignment *∪ *traceback*(*u*, *v*) {*traceback *returns the alignment obtained by tracing back from it's argument}

6:    Mark the rows and columns of *L *corresponding to the residues returned in the current alignment *done*.

7:    Compute *highest *= *max*_*i*, *j *_*L*_*i*, *j *_such that *i *or *j *is not marked *done*.

8:    Compute (*u*, *v*) = *arg *max_*i*, *j *_*L*_*i*, *j *_such that *i *or *j *is not marked *done*.

9: end while

as a set of points in 3D. It is well known, that such a set of point can be matched under rigid transformation using graph matching techniques.

Let NstrA
 MathType@MTEF@5@5@+=feaafiart1ev1aaatCvAUfKttLearuWrP9MDH5MBPbIqV92AaeXatLxBI9gBaebbnrfifHhDYfgasaacH8akY=wiFfYdH8Gipec8Eeeu0xXdbba9frFj0=OqFfea0dXdd9vqai=hGuQ8kuc9pgc9s8qqaq=dirpe0xb9q8qiLsFr0=vr0=vr0dc8meaabaqaciaacaGaaeqabaqabeGadaaakeaacqWGobGtdaqhaaWcbaGaem4CamNaemiDaqNaemOCaihabaGaemyqaeeaaaaa@3356@(*i*) and NstrB
 MathType@MTEF@5@5@+=feaafiart1ev1aaatCvAUfKttLearuWrP9MDH5MBPbIqV92AaeXatLxBI9gBaebbnrfifHhDYfgasaacH8akY=wiFfYdH8Gipec8Eeeu0xXdbba9frFj0=OqFfea0dXdd9vqai=hGuQ8kuc9pgc9s8qqaq=dirpe0xb9q8qiLsFr0=vr0=vr0dc8meaabaqaciaacaGaaeqabaqabeGadaaakeaacqWGobGtdaqhaaWcbaGaem4CamNaemiDaqNaemOCaihabaGaemOqaieaaaaa@3358@(*j*) be the 2 neighborhoods to be compared. We construct two graphs *G*^*A *^= (NstrA
 MathType@MTEF@5@5@+=feaafiart1ev1aaatCvAUfKttLearuWrP9MDH5MBPbIqV92AaeXatLxBI9gBaebbnrfifHhDYfgasaacH8akY=wiFfYdH8Gipec8Eeeu0xXdbba9frFj0=OqFfea0dXdd9vqai=hGuQ8kuc9pgc9s8qqaq=dirpe0xb9q8qiLsFr0=vr0=vr0dc8meaabaqaciaacaGaaeqabaqabeGadaaakeaacqWGobGtdaqhaaWcbaGaem4CamNaemiDaqNaemOCaihabaGaemyqaeeaaaaa@3356@(*i*), NstrA
 MathType@MTEF@5@5@+=feaafiart1ev1aaatCvAUfKttLearuWrP9MDH5MBPbIqV92AaeXatLxBI9gBaebbnrfifHhDYfgasaacH8akY=wiFfYdH8Gipec8Eeeu0xXdbba9frFj0=OqFfea0dXdd9vqai=hGuQ8kuc9pgc9s8qqaq=dirpe0xb9q8qiLsFr0=vr0=vr0dc8meaabaqaciaacaGaaeqabaqabeGadaaakeaacqWGobGtdaqhaaWcbaGaem4CamNaemiDaqNaemOCaihabaGaemyqaeeaaaaa@3356@(*i*) × NstrA
 MathType@MTEF@5@5@+=feaafiart1ev1aaatCvAUfKttLearuWrP9MDH5MBPbIqV92AaeXatLxBI9gBaebbnrfifHhDYfgasaacH8akY=wiFfYdH8Gipec8Eeeu0xXdbba9frFj0=OqFfea0dXdd9vqai=hGuQ8kuc9pgc9s8qqaq=dirpe0xb9q8qiLsFr0=vr0=vr0dc8meaabaqaciaacaGaaeqabaqabeGadaaakeaacqWGobGtdaqhaaWcbaGaem4CamNaemiDaqNaemOCaihabaGaemyqaeeaaaaa@3356@(*i*)) and *G*^*B *^= (NstrB
 MathType@MTEF@5@5@+=feaafiart1ev1aaatCvAUfKttLearuWrP9MDH5MBPbIqV92AaeXatLxBI9gBaebbnrfifHhDYfgasaacH8akY=wiFfYdH8Gipec8Eeeu0xXdbba9frFj0=OqFfea0dXdd9vqai=hGuQ8kuc9pgc9s8qqaq=dirpe0xb9q8qiLsFr0=vr0=vr0dc8meaabaqaciaacaGaaeqabaqabeGadaaakeaacqWGobGtdaqhaaWcbaGaem4CamNaemiDaqNaemOCaihabaGaemOqaieaaaaa@3358@(*j*), NstrB
 MathType@MTEF@5@5@+=feaafiart1ev1aaatCvAUfKttLearuWrP9MDH5MBPbIqV92AaeXatLxBI9gBaebbnrfifHhDYfgasaacH8akY=wiFfYdH8Gipec8Eeeu0xXdbba9frFj0=OqFfea0dXdd9vqai=hGuQ8kuc9pgc9s8qqaq=dirpe0xb9q8qiLsFr0=vr0=vr0dc8meaabaqaciaacaGaaeqabaqabeGadaaakeaacqWGobGtdaqhaaWcbaGaem4CamNaemiDaqNaemOCaihabaGaemOqaieaaaaa@3358@(*j*) × NstrB
 MathType@MTEF@5@5@+=feaafiart1ev1aaatCvAUfKttLearuWrP9MDH5MBPbIqV92AaeXatLxBI9gBaebbnrfifHhDYfgasaacH8akY=wiFfYdH8Gipec8Eeeu0xXdbba9frFj0=OqFfea0dXdd9vqai=hGuQ8kuc9pgc9s8qqaq=dirpe0xb9q8qiLsFr0=vr0=vr0dc8meaabaqaciaacaGaaeqabaqabeGadaaakeaacqWGobGtdaqhaaWcbaGaem4CamNaemiDaqNaemOCaihabaGaemOqaieaaaaa@3358@(*j*)). Also, consider the weighting functions *w*_1 _and *w*_2 _defined on the edges of the graphs *G*^*A *^and *G*^*B *^as *w*_1_(*e*) = ||**x**_*p *_- **x**_*q*_||, (**x**_*p*_, **x**_*q*_) = *e *∈ *E*(*G*^*A*^) and *w*_2_(*e'*) = ||**x**_*r *_- **x**_*s*_||, (**x**_*r*_, **x**_*s*_) = *e' *∈ *E*(*G*^*B*^). Two edges *e *and *e' *from each graph are said to compatible if (*distT *- |*w*_1_(*e*) - *w*_2 _(*e'*)|) ≥ 0, incompatible otherwise. A mapping *g *of vertices from *G*^*A *^to *G*^*B *^is called isomorphism if, for *v*_1_, *v*_2 _∈ *G*^*A*^, the edges (*v*_1_, *v*_2_) and (*g*(*v*_1_), *g*(*v*_2_)) are compatible. The problem of finding a maximal match between the two neighborhoods is same as finding the maximal subgraph of *G*^*A *^having an isomorphic subgraph in *G*^*B*^.

This problem of finding a *maximal common subgraph *between *G*^*A *^and *G*^*B *^is solved using the algorithm described in [24], with the added constraint that the central residues of both the neighborhoods NstrA
 MathType@MTEF@5@5@+=feaafiart1ev1aaatCvAUfKttLearuWrP9MDH5MBPbIqV92AaeXatLxBI9gBaebbnrfifHhDYfgasaacH8akY=wiFfYdH8Gipec8Eeeu0xXdbba9frFj0=OqFfea0dXdd9vqai=hGuQ8kuc9pgc9s8qqaq=dirpe0xb9q8qiLsFr0=vr0=vr0dc8meaabaqaciaacaGaaeqabaqabeGadaaakeaacqWGobGtdaqhaaWcbaGaem4CamNaemiDaqNaemOCaihabaGaemyqaeeaaaaa@3356@(*i*) and NstrB
 MathType@MTEF@5@5@+=feaafiart1ev1aaatCvAUfKttLearuWrP9MDH5MBPbIqV92AaeXatLxBI9gBaebbnrfifHhDYfgasaacH8akY=wiFfYdH8Gipec8Eeeu0xXdbba9frFj0=OqFfea0dXdd9vqai=hGuQ8kuc9pgc9s8qqaq=dirpe0xb9q8qiLsFr0=vr0=vr0dc8meaabaqaciaacaGaaeqabaqabeGadaaakeaacqWGobGtdaqhaaWcbaGaem4CamNaemiDaqNaemOCaihabaGaemOqaieaaaaa@3358@(*j*) be matched. The parameter *distT *is used to control the allowed deviation in distances. This value is kept small (typically 1 Å) as the neighborhoods are required to be matched very accurately. The algorithm scales exponentially with the neighborhood size. All results are reported for neighborhood size of 6. The algorithm becomes very slow for neighborhood sizes above 15. In the next section, a faster algorithm, which can handle larger neighborhoods, albeit with a slight loss of accuracy, is described.

### 0.10 Aligning sequence neighborhoods

Sequence neighborhoods, like structure neighborhoods, are sets of points in 3D space. Thus, the problem can be solved using the algorithm mentioned above. However, there is natural topological connection between them. We utilize this fact to derive a more efficient, though less exact, algorithm for matching sequence neighborhoods. Our algorithm is based on *spectral graph matching *technique described first in [9]. Define the graphs *G*^*A *^and *G*^*B *^as in the previous section, with NstrA
 MathType@MTEF@5@5@+=feaafiart1ev1aaatCvAUfKttLearuWrP9MDH5MBPbIqV92AaeXatLxBI9gBaebbnrfifHhDYfgasaacH8akY=wiFfYdH8Gipec8Eeeu0xXdbba9frFj0=OqFfea0dXdd9vqai=hGuQ8kuc9pgc9s8qqaq=dirpe0xb9q8qiLsFr0=vr0=vr0dc8meaabaqaciaacaGaaeqabaqabeGadaaakeaacqWGobGtdaqhaaWcbaGaem4CamNaemiDaqNaemOCaihabaGaemyqaeeaaaaa@3356@(*i*) and NstrB
 MathType@MTEF@5@5@+=feaafiart1ev1aaatCvAUfKttLearuWrP9MDH5MBPbIqV92AaeXatLxBI9gBaebbnrfifHhDYfgasaacH8akY=wiFfYdH8Gipec8Eeeu0xXdbba9frFj0=OqFfea0dXdd9vqai=hGuQ8kuc9pgc9s8qqaq=dirpe0xb9q8qiLsFr0=vr0=vr0dc8meaabaqaciaacaGaaeqabaqabeGadaaakeaacqWGobGtdaqhaaWcbaGaem4CamNaemiDaqNaemOCaihabaGaemOqaieaaaaa@3358@(*j*) replaced with NseqA
 MathType@MTEF@5@5@+=feaafiart1ev1aaatCvAUfKttLearuWrP9MDH5MBPbIqV92AaeXatLxBI9gBaebbnrfifHhDYfgasaacH8akY=wiFfYdH8Gipec8Eeeu0xXdbba9frFj0=OqFfea0dXdd9vqai=hGuQ8kuc9pgc9s8qqaq=dirpe0xb9q8qiLsFr0=vr0=vr0dc8meaabaqaciaacaGaaeqabaqabeGadaaakeaacqWGobGtdaqhaaWcbaGaem4CamNaemyzauMaemyCaehabaGaemyqaeeaaaaa@3336@(*i*) and NseqB
 MathType@MTEF@5@5@+=feaafiart1ev1aaatCvAUfKttLearuWrP9MDH5MBPbIqV92AaeXatLxBI9gBaebbnrfifHhDYfgasaacH8akY=wiFfYdH8Gipec8Eeeu0xXdbba9frFj0=OqFfea0dXdd9vqai=hGuQ8kuc9pgc9s8qqaq=dirpe0xb9q8qiLsFr0=vr0=vr0dc8meaabaqaciaacaGaaeqabaqabeGadaaakeaacqWGobGtdaqhaaWcbaGaem4CamNaemyzauMaemyCaehabaGaemOqaieaaaaa@3338@(*j*), respectively. Define the weight functions *w*_1 _and *w*_2 _as *w*_1_(*e*) = e−Dpqα
 MathType@MTEF@5@5@+=feaafiart1ev1aaatCvAUfKttLearuWrP9MDH5MBPbIqV92AaeXatLxBI9gBaebbnrfifHhDYfgasaacH8akY=wiFfYdH8Gipec8Eeeu0xXdbba9frFj0=OqFfea0dXdd9vqai=hGuQ8kuc9pgc9s8qqaq=dirpe0xb9q8qiLsFr0=vr0=vr0dc8meaabaqaciaacaGaaeqabaqabeGadaaakeaacqWGLbqzdaahaaWcbeqaamaalaaabaGaeyOeI0Iaemiraq0aaSbaaWqaaiabdchaWjabdghaXbqabaaaleaaiiGacqWFXoqyaaaaaaaa@34EC@, *D*_*pq *_= ||**x**_*p *_- **x**_*q*_||, (**x**_*p*_, **x**_*q*_) = *e *∈ *E*(*G*^*A*^) and *w*_2 _(*e'*) = e−Drsα
 MathType@MTEF@5@5@+=feaafiart1ev1aaatCvAUfKttLearuWrP9MDH5MBPbIqV92AaeXatLxBI9gBaebbnrfifHhDYfgasaacH8akY=wiFfYdH8Gipec8Eeeu0xXdbba9frFj0=OqFfea0dXdd9vqai=hGuQ8kuc9pgc9s8qqaq=dirpe0xb9q8qiLsFr0=vr0=vr0dc8meaabaqaciaacaGaaeqabaqabeGadaaakeaacqWGLbqzdaahaaWcbeqaamaalaaabaGaeyOeI0Iaemiraq0aaSbaaWqaaiabdkhaYjabdohaZbqabaaaleaaiiGacqWFXoqyaaaaaaaa@34F4@, *D*_*rs *_= ||**x**_*r *_- **x**_*s*_||, (**x**_*r*_, **x**_*s*_) = *e' *∈ *E*(*G*^*B*^), where *α *is a parameter governing the rate of decay of the weight functions. Let N
 MathType@MTEF@5@5@+=feaafiart1ev1aaatCvAUfKttLearuWrP9MDH5MBPbIqV92AaeXatLxBI9gBamrtHrhAL1wy0L2yHvtyaeHbnfgDOvwBHrxAJfwnaebbnrfifHhDYfgasaacH8akY=wiFfYdH8Gipec8Eeeu0xXdbba9frFj0=OqFfea0dXdd9vqai=hGuQ8kuc9pgc9s8qqaq=dirpe0xb9q8qiLsFr0=vr0=vr0dc8meaabaqaciaacaGaaeqabaWaaeGaeaaakeaaimaacqWFneVtaaa@383B@^*A *^and N
 MathType@MTEF@5@5@+=feaafiart1ev1aaatCvAUfKttLearuWrP9MDH5MBPbIqV92AaeXatLxBI9gBamrtHrhAL1wy0L2yHvtyaeHbnfgDOvwBHrxAJfwnaebbnrfifHhDYfgasaacH8akY=wiFfYdH8Gipec8Eeeu0xXdbba9frFj0=OqFfea0dXdd9vqai=hGuQ8kuc9pgc9s8qqaq=dirpe0xb9q8qiLsFr0=vr0=vr0dc8meaabaqaciaacaGaaeqabaWaaeGaeaaakeaaimaacqWFneVtaaa@383B@^*B *^be adjacency matrices of *G*^*A *^and *G*^*B*^. We are interested in finding a mapping between vertices of *G*^*A *^and *G*^*B*^, or equivalently a permutation of bases of N
 MathType@MTEF@5@5@+=feaafiart1ev1aaatCvAUfKttLearuWrP9MDH5MBPbIqV92AaeXatLxBI9gBamrtHrhAL1wy0L2yHvtyaeHbnfgDOvwBHrxAJfwnaebbnrfifHhDYfgasaacH8akY=wiFfYdH8Gipec8Eeeu0xXdbba9frFj0=OqFfea0dXdd9vqai=hGuQ8kuc9pgc9s8qqaq=dirpe0xb9q8qiLsFr0=vr0=vr0dc8meaabaqaciaacaGaaeqabaWaaeGaeaaakeaaimaacqWFneVtaaa@383B@^*A *^or N
 MathType@MTEF@5@5@+=feaafiart1ev1aaatCvAUfKttLearuWrP9MDH5MBPbIqV92AaeXatLxBI9gBamrtHrhAL1wy0L2yHvtyaeHbnfgDOvwBHrxAJfwnaebbnrfifHhDYfgasaacH8akY=wiFfYdH8Gipec8Eeeu0xXdbba9frFj0=OqFfea0dXdd9vqai=hGuQ8kuc9pgc9s8qqaq=dirpe0xb9q8qiLsFr0=vr0=vr0dc8meaabaqaciaacaGaaeqabaWaaeGaeaaakeaaimaacqWFneVtaaa@383B@^*B*^.

For calculating the best permutation, we calculate the eigenvalue decomposition of the nearness matrices as N
 MathType@MTEF@5@5@+=feaafiart1ev1aaatCvAUfKttLearuWrP9MDH5MBPbIqV92AaeXatLxBI9gBamrtHrhAL1wy0L2yHvtyaeHbnfgDOvwBHrxAJfwnaebbnrfifHhDYfgasaacH8akY=wiFfYdH8Gipec8Eeeu0xXdbba9frFj0=OqFfea0dXdd9vqai=hGuQ8kuc9pgc9s8qqaq=dirpe0xb9q8qiLsFr0=vr0=vr0dc8meaabaqaciaacaGaaeqabaWaaeGaeaaakeaaimaacqWFneVtaaa@383B@ = ∑k=1nλkζkζiT
 MathType@MTEF@5@5@+=feaafiart1ev1aaatCvAUfKttLearuWrP9MDH5MBPbIqV92AaeXatLxBI9gBaebbnrfifHhDYfgasaacH8akY=wiFfYdH8Gipec8Eeeu0xXdbba9frFj0=OqFfea0dXdd9vqai=hGuQ8kuc9pgc9s8qqaq=dirpe0xb9q8qiLsFr0=vr0=vr0dc8meaabaqaciaacaGaaeqabaqabeGadaaakeaadaaeWaqaaGGaciab=T7aSnaaBaaaleaacqWGRbWAaeqaaOGae8NTdO3aaSbaaSqaaiabdUgaRbqabaGccqWF2oGEdaqhaaWcbaGaemyAaKgabaGaemivaqfaaaqaaiabdUgaRjabg2da9iabigdaXaqaaiabd6gaUbqdcqGHris5aaaa@3E6B@. It can be easily shown that if the rows and columns (or bases) of a matrix is permuted by certain permutation, its eigenvalues remain the same and the bases of its eigenvectors are also permuted by the same permutation and vice versa. Thus, considering only the eigenvector corresponding to the highest eigenvalue, for two similar matrices, the difference between the permuted eigenvectors should be minimal. We use this fact to define a similarity measure between the bases of the two matrices, and hence between the residues of the two neighborhoods.

Let **f**^*A *^and **f**^*B *^be the eigenvectors corresponding to the highest eigenvalues of N
 MathType@MTEF@5@5@+=feaafiart1ev1aaatCvAUfKttLearuWrP9MDH5MBPbIqV92AaeXatLxBI9gBamrtHrhAL1wy0L2yHvtyaeHbnfgDOvwBHrxAJfwnaebbnrfifHhDYfgasaacH8akY=wiFfYdH8Gipec8Eeeu0xXdbba9frFj0=OqFfea0dXdd9vqai=hGuQ8kuc9pgc9s8qqaq=dirpe0xb9q8qiLsFr0=vr0=vr0dc8meaabaqaciaacaGaaeqabaWaaeGaeaaakeaaimaacqWFneVtaaa@383B@^*A *^and N
 MathType@MTEF@5@5@+=feaafiart1ev1aaatCvAUfKttLearuWrP9MDH5MBPbIqV92AaeXatLxBI9gBamrtHrhAL1wy0L2yHvtyaeHbnfgDOvwBHrxAJfwnaebbnrfifHhDYfgasaacH8akY=wiFfYdH8Gipec8Eeeu0xXdbba9frFj0=OqFfea0dXdd9vqai=hGuQ8kuc9pgc9s8qqaq=dirpe0xb9q8qiLsFr0=vr0=vr0dc8meaabaqaciaacaGaaeqabaWaaeGaeaaakeaaimaacqWFneVtaaa@383B@^*B*^, respectively. We define the similarity *sim*(*i*, *j*) between residue *i *of neighborhood NseqA
 MathType@MTEF@5@5@+=feaafiart1ev1aaatCvAUfKttLearuWrP9MDH5MBPbIqV92AaeXatLxBI9gBaebbnrfifHhDYfgasaacH8akY=wiFfYdH8Gipec8Eeeu0xXdbba9frFj0=OqFfea0dXdd9vqai=hGuQ8kuc9pgc9s8qqaq=dirpe0xb9q8qiLsFr0=vr0=vr0dc8meaabaqaciaacaGaaeqabaqabeGadaaakeaacqWGobGtdaqhaaWcbaGaem4CamNaemyzauMaemyCaehabaGaemyqaeeaaaaa@3336@(*i*) and residue NseqB
 MathType@MTEF@5@5@+=feaafiart1ev1aaatCvAUfKttLearuWrP9MDH5MBPbIqV92AaeXatLxBI9gBaebbnrfifHhDYfgasaacH8akY=wiFfYdH8Gipec8Eeeu0xXdbba9frFj0=OqFfea0dXdd9vqai=hGuQ8kuc9pgc9s8qqaq=dirpe0xb9q8qiLsFr0=vr0=vr0dc8meaabaqaciaacaGaaeqabaqabeGadaaakeaacqWGobGtdaqhaaWcbaGaem4CamNaemyzauMaemyCaehabaGaemOqaieaaaaa@3338@(*j*) of neighborhood 2 as:

*sim*(*i*, *j*) = *T' *- |fi1
 MathType@MTEF@5@5@+=feaafiart1ev1aaatCvAUfKttLearuWrP9MDH5MBPbIqV92AaeXatLxBI9gBaebbnrfifHhDYfgasaacH8akY=wiFfYdH8Gipec8Eeeu0xXdbba9frFj0=OqFfea0dXdd9vqai=hGuQ8kuc9pgc9s8qqaq=dirpe0xb9q8qiLsFr0=vr0=vr0dc8meaabaqaciaacaGaaeqabaqabeGadaaakeaaieqacqWFMbGzdaqhaaWcbaGaemyAaKgabaGaeGymaedaaaaa@307F@ - fj2
 MathType@MTEF@5@5@+=feaafiart1ev1aaatCvAUfKttLearuWrP9MDH5MBPbIqV92AaeXatLxBI9gBaebbnrfifHhDYfgasaacH8akY=wiFfYdH8Gipec8Eeeu0xXdbba9frFj0=OqFfea0dXdd9vqai=hGuQ8kuc9pgc9s8qqaq=dirpe0xb9q8qiLsFr0=vr0=vr0dc8meaabaqaciaacaGaaeqabaqabeGadaaakeaaieqacqWFMbGzdaqhaaWcbaGaemOAaOgabaGaeGOmaidaaaaa@3083@|, 1 ≤ *i*, *j *≤ *k *   (3)

Here, *T' *is the maximum allowed difference in the eigenvector values. *T' *can be used to control the precision of the returned match. We are interested in equivalences that maximize the sum total score of similarities between corresponding residues. Also, since there in a natural sequence connectivity between residues of the neighborhood, we can use the greedy fragment pair search technique described in section 0.8. The equivalences thus obtained are returned as the optimal alignment between the sequence neighborhoods.

## Availability and requirements

Project name: Matchprot

Project home page: 

Operating system(s): Platform independent

Programming language: C/C++

Other requirements: Web browser

License: Web access is free for all.

Any restrictions to use by non-academics: none

## Authors' contributions

S.B. and C.B. devised the algorithm; S.B. implemented it. N.C. helped in analyzing the results and interpreting the biological implications.

## Supplementary Material

Additional File 1 Detailed results. This file contains RMSD and length of alignments for all protein pairs in all the benchmark datasets, obtained with Matchprot, DALI, CE, and SSM. Click here for file
