# Supplementary material for: Comparison of protein structures by growing neighborhood alignments
Source: BMC Bioinformatics. 2007 Mar 6;8:77. doi: 10.1186/1471-2105-8-77 (PMC1828169; doi:10.1186/1471-2105-8-77)
Supplement: Additional File 1 — Detailed results. This file contains RMSD and length of alignments for all protein pairs in all the benchmark datasets, obtained with Matchprot, DALI, CE, and SSM. [file 1471-2105-8-77-S1.pdf]

Default Parameters

Parameter name	*Value (sequence nbhd.)*	*Value (structure nbhd.)*
Neighborhood Size	17	6
Tolerance in distance between residues (*T*) (Eqn 1)	5.0	5.0
Cutoff on length of substructure alignments (*LenCutoff*)	14	5
Gap penalty for final Greedy Fragment Pair Search (*gap2*)	0.1	0.1
Cluster Threshold for clustering transformations (*ClustThreshold*)	1.0	1.0
Cutoff on Ratio of length diff. And RMSD diff. (*LRCutoff*)	30	30
Threshold on difference in distance (*distT*)	--	1.0
Alpha for calculating weights of substructure graphs (*alpha*)	10.0	--
Threshold on difference in eigenvector values (*T'*)	0.1	--
Gap penalty for the sequence neighborhood alignment (*gap*)	0.05	--

Comparison with DALI

Fisher's Dataset

Sequence Neighborhood based program:

PDBid1-PDBid2	Proposed Program (Lali / RMSD)	Dali (Lali / RMSD / Zscore)	Good / Bad / Level
1dxtB - 1hbg	117 / 1.767981	135 / 2 / 17.8	L
1cpcL - 1colA	89 / 2.345877	114 / 3.6 / 5.8	L
1c2rA - 1ycc	94 / 1.824597	96 / 1.6 / 13.5	B
2mtaC - 1ycc	74 / 1.907191	80 / 2.1 / 6.2	L
1bbhA - 2ccyA	116 / 1.603838	125 / 2 / 16.2	L
1bgeB - 2gmfA	85 / 2.480859	94 / 3.3 / 6.6	L
1rcb - 2gmfA	76 / 2.211365	82 / 3.3 / 5.8	L
1aep - 256bA	71 / 1.938330	74 1.8 / 5.7	B
1osa - 4cpv	66 / 1.274414	67 / 1.4 / 9.4	L
2sas - 2scpA	122 / 2.229376	168 / 3.6 / 14.9	L
1hom - 1lfb	54 / 1.522779	56 / 1.9 / 11.5	L
1lgaA - 2cyp	242 / 1.768907	261 / 2.4 / 27.5	L
2hpdA - 2cpp	307 / 2.487783	374 / 3.5 / 29.5	L
1fc1A - 2fb4H	109 / 1.894847	101 / 2.9 / 13.7	G
2fbjL - 8fabB	180 / 1.810727	194 / 2.3 / 18.4	L'
1cid - 2rhe	91 / 2.059754	97 / 3.2 / 8.3	L'
1pfc - 3hlaB	80 / 2.259232	88 / 2.8 / 7.6	L
1ten - 3hhrB	84 / 1.580910	86 / 1.9 / 11.5	L
1tlk - 2rhe	87 / 1.580035	89 / 2 / 11.3	L'
3cd4 - 2rhe	88 / 1.381726	94 / 2.6 / 12.1	L
3hlaB - 2rhe	70 / 2.269583	75 / 3 / 5.4	L
1aaj - 1paz	80 / 1.564230	80 / 1.7 / 9.7	G
2afnA - 1aozA	233 / 2.098379	248 / 2.6 / 18.5	L

2azaA - 1paz	72 / 2.463617	81 / 2.5 / 6.4	L
4sbvA - 2tbvA	155 / 1.482077	162 / 2.1 / 18.3	L
1bbt1 - 2plv1	147 / 2.356716	168 / 2.6 / 10.8	L
1sacA - 2ayh	127 / 2.592137	133 / 3 / 8	L
1ltsD - 1bovA	63 / 2.317266	67 / 1.9 / 7.6	B
1tie - 4fgf	105 / 2.209304	114 / 3.1 / 9.8	L
8i1b - 4fgf	110 / 1.915536	118 / 2.5 / 14.1	L
1arb - 5ptp	162 / 2.171203	189 / 2.9 / 17.6	L
2sga - 5ptp	132 / 1.971517	147 / 2.7 / 13.8	L
2snv - 5ptp	117 / 2.368816	131 / 3.1 / 9.2	L
1mdc - 1ifc	127 / 2.512997	- / - / -	G
1mup - 1rbp	121 / 2.120736	140 / 2.9 / 13.9	L
2sim - 1nsbA	262 / 2.727954	222 / 3.8 / 10.7	G
1cauB - 1cauA	153 / 2.057075	163 / 2.2 / 17.6	L
2omf - 2por	240 / 2.259293	261 / 2.7 / 21.5	L
1chrA - 2mnr	340 / 1.647549	347 / 1.9 / 40.9	L
2mnr - 4enl	234 / 2.363370	285 / 3.4 / 21.6	L
3rubL - 6xia	147 / 2.609368	206 / 4.1 / 7.9	L
1crl - 1ede	160 / 2.503302	211 / 3.5 / 12.1	L
1tahA - 1tca	172 / 2.020544	188 / 2.5 / 14.9	L
1aba - 1ego	69 / 1.886953	72 / 2.2 / 8.6	L
1dsbA - 2trxA	69 / 2.141120	84 / 2.9 / 5.3	L
1gp1A - 2trxA	89 / 2.308867	98 / 2.6 / 8	L
1atnA - 1atr	264 / 2.099867	292 / 3 / 25.1	L
1hrhA - 1rnh	108 / 1.545015	114 / 2 / 14.9	L

3chy - 2fox	91 / 2.370168	103 / 3 / 6.8	L
2ak3A - 1gky	125 / 2.537671	149 / 3 / 10.8	L
1gky - 3adk	124 / 2.093791	154 / 3 / 12.4	L
2cmd - 6ldh	267 / 2.034366	286 / 2.5 / 30.2	L
1eaf - 4cla	144 / 2.284240	174 / 2.6 / 17.4	L
2gbp - 2liv	167 / 2.600682	260 / 6.7 / 15.7	L
1mioC - 2minB	304 / 2.547204	410 / 3.6 / 26.3	L
2pia - 1fnb	186 / 2.057759	216 / 2.5 / 21.2	L
1gal - 3cox	372 / 2.337642	401 / 3.1 / 25.7	L
1npx - 3grs	333 / 2.251019	395 / 3.5 / 30.6	L
1fxiA - 1ubq	54 / 2.183049	60 / 2.6 / 4	L
1cewI - 1molA	68 / 1.804976	81 / 2.3 / 9.3	L
1stfI - 1molA	77 / 1.870025	85 / 1.9 / 10.7	L
2pna - 1shaA	81 / 1.856416	92 / 2.6 / 9.4	L
2sarA - 9rnt	61 / 2.485668	71 / 3.2 / 5	L
1onc - 7rsa	90 / 2.201218	97 / 1.9 / 13.6	B
5fd1 - 2fxb	52 / 1.684135	57 / 2.6 / 3.7	L
2hhmA - 1fbpA	198 / 2.395615	224 / 2.8 / 19.7	L
1hip - 2hipA	66 / 1.716334	67 / 1.8 / 10	L
1isuA - 2hipA	54 / 1.692477	58 / 2.3 / 7.5	L

Structure Neighborhood based program:

PDBid1 - PDBid2	Proposed Program (Lali / RMSD)	DALI (Lali / rmsd / zscore)	Good / Bad / Level
1dxtB - 1hbg	133 / 1.618052	135 / 2 / 17.8	L
1cpcL - 1colA	91 / 2.453736	114 / 3.6 / 5.8	L
1c2rA - 1ycc	95 / 1.362028	96 / 1.6 / 13.5	L
2mtaC - 1ycc	78 / 1.941872	80 / 2.1 / 6.2	L
1bbhA - 2ccyA	121 / 1.764434	125 / 2 / 16.2	L
1bgeB - 2gmfA	87 / 2.225433	94 / 3.3 / 6.6	L
1rcb - 2gmfA	77 / 1.879182	82 / 3.3 / 5.8	L
1aep - 256bA	64 / 2.117783	74 / 1.8 / 5.7	B
1osa - 4cpv	65 / 1.526141	67 / 1.4 / 9.4	B
2sas - 2scpA	138 / 2.231157	168 / 3.6 / 14.9	L
1hom - 1lfb	52 / 1.281308	56 / 1.9 / 11.5	L
1lgaA - 2cyp	238 / 2.192340	261 / 2.4 / 27.5	L
2hpdA - 2cpp	283 / 2.585693	374 / 3.5 / 29.5	L
1fc1A - 2fb4H	112 / 1.892321	101 / 2.9 / 13.7	G
2fbjL - 8fabB	185 / 1.735389	194 / 2.3 / 18.4	L
1cid - 2rhe	91 / 2.068938	97 / 3.2 / 8.3	L
1pfc - 3hlaB	85 / 2.230198	88 / 2.8 / 7.6	L
1ten - 3hhrB	82 / 1.392378	86 / 1.9 / 11.5	L
1tlk - 2rhe	84 / 1.347123	89 / 2 / 11.3	L
3cd4 - 2rhe	88 / 1.596862	94 / 2.6 / 12.1	L
3hlaB - 2rhe	68 / 2.266716	75 / 3 / 5.4	L
1aaj - 1paz	80 / 1.488212	80 / 1.7 / 9.7	G
2afnA - 1aozA	227 / 2.134964	248 / 2.6 / 18.5	L

2azaA - 1paz	79 / 2.208511	81 / 2.5 / 6.4	L
4sbvA - 2tbvA	154 / 1.573555	162 / 2.1 / 18.3	L
1bbt1 - 2plv1	156 / 2.045998	168 / 2.6 / 10.8	L
1sacA - 2ayh	139 / 2.453903	133 / 3 / 8	G
1ltsD - 1bovA	67 / 1.769267	67 / 1.9 / 7.6	G
1tie - 4fgf	106 / 2.279553	114 / 3.1 / 9.8	L
8i1b - 4fgf	110 / 1.901800	118 / 2.5 / 14.1	L
1arb - 5ptp	168 / 1.986419	189 / 2.9 / 17.6	L
2sga - 5ptp	129 / 1.833044	147 / 2.7 / 13.8	L
2snv - 5ptp	96 / 2.290828	131 / 3.1 / 9.2	L
1mdc - 1ifc	- / -	- / - / -	L
1mup - 1rbp	122 / 2.009872	140 / 2.9 / 13.9	L
2sim - 1nsbA	262 / 2.632144	222 / 3.8 / 10.7	G
1cauB - 1cauA	152 / 1.738604	163 / 2.2 / 17.6	L
2omf - 2por	235 / 2.467897	261 / 2.7 / 21.5	L
1chrA - 2mnr	335 / 1.664985	347 / 1.9 / 40.9	L
2mnr - 4enl	230 / 2.517675	285 / 3.4 / 21.6	L
3rubL - 6xia	152 / 2.796273	206 / 4.1 / 7.9	L
1crl - 1ede	174 / 2.491762	211 / 3.5 / 12.1	L
1tahA - 1tca	168 / 2.416043	188 / 2.5 / 14.9	L
1aba - 1ego	69 / 2.000439	72 / 2.2 / 8.6	L
1dsbA - 2trxA	76 / 1.839218	84 / 2.9 / 5.3	L
1gp1A - 2trxA	86 / 1.886982	98 / 2.6 / 8	L
1atnA - 1atr	260 / 2.181434	292 / 3 / 25.1	L
1hrhA - 1rnh	108 / 1.511972	114 / 2 / 14.9	L

3chy - 2fox	91 / 2.430114	103 / 3 / 6.8	L
2ak3A - 1gky	135 / 2.352513	149 / 3 / 10.8	L
1gky - 3adk	141 / 2.253797	154 / 3 / 12.4	L
2cmd - 6ldh	274 / 2.056520	286 / 2.5 / 30.2	L
1eaf - 4cla	160 / 2.022917	174 / 2.6 / 17.4	L
2gbp - 2liv	169 / 2.544403	260 / 6.7 / 15.7	L
1mioC - 2minB	318 / 2.330087	410 / 3.6 / 26.3	L
2pia - 1fnb	195 / 1.855691	216 / 2.5 / 21.2	L
1gal - 3cox	366 / 2.119449	401 / 3.1 / 25.7	L
1npx - 3grs	345 / 2.197732	395 / 3.5 / 30.6	L
1fxiA - 1ubq	56 / 2.163154	60 / 2.6 / 4	L
1cewI - 1molA	79 / 1.944987	81 / 2.3 / 9.3	L
1stfI - 1molA	82 / 1.852846	85 / 1.9 / 10.7	L
2pna - 1shaA	83 / 1.941611	92 / 2.6 / 9.4	L
2sarA - 9rnt	64 / 2.298871	71 / 3.2 / 5	L
1onc - 7rsa	93 / 1.296087	97 / 1.9 / 13.6	L
5fd1 - 2fxb	55 / 1.879364	57 / 2.6 / 3.7	L
2hhmA - 1fbpA	187 / 2.103918	224 / 2.8 / 19.7	L
1hip - 2hipA	65 / 1.462879	67 / 1.8 / 10	L
1isuA - 2hipA	53 / 1.497409	58 / 2.3 / 7.5	L

Novotny's Dataset

Sequence Neighborhood based program:

PDBid1 - PDBid2	Proposed Program (Lali / RMSD)	DALI (Lali / RMSD / Zscore)	Good / Bad / Level
**1.10.164**			
1aq6A - 1c3uA	52 / 2.552922	60 / 3.6 / 1.0	L
1aq6A - 1fezA	161 / 1.993285	201 / 4.0 / 16.2	L
1aq6A - 1jud	217 / 1.304811	218 / 1.3 / 30.5	L
1aq6A - 1zrn	217 / 1.292294	218 / 1.3 / 30.9	L
1c3uA - 1fezA	55 / 2.231143	56 / 3.8 / 2.4	L
1c3uA - 1jud	15 / 0.953262	58 / 3.3 / 3.6	L
1c3uA - 1zrn	78 / 2.630662	59 / 3.1 / 3.3	G
1fezA - 1jud	157 / 1.974788	188 / 3.4 / 17.3	L
1fezA - 1zrn	163 / 2.162118	189 / 3.4 / 17.0	L
1jud - 1zrn	220 / 0.265135	220 / 0.3 / 39.1	L
**1.10.40**			
1rlr - 1yfm	95 / 3.074015	49 / 3.6 / 2.2	G
1rlr - 1furA	85 / 2.771679	48 / 3.7 / 2.3	G
1rlr - 1auwA	85 / 2.734123	60 / 3.3 / 3.1	G
1rlr - 1jswA	86 / 2.680286	63 / 4.6 / 1.2	G
1rlr - 1hylA	62 / 2.972107	- / - / -	G
1rlr - 1i0aA	79 / 2.859903	64 / 3.4 / 2.9	G
1yfm - 1furA	446 / 1.007248	446 / 1.0 / 50.4	L
1yfm - 1auwA	347 / 2.131289	389 / 2.9 / 31.4	L
1yfm - 1jswA	423 / 1.807071	443 / 1.9 / 48.9	L
1yfm - 1hylA	55 / 2.680767	- / - / -	G
1yfm - 1i0aA	350 / 1.964233	389 / 2.9 / 31.7	L

1furA - 1auwA	342 / 1.942800	386 / 3.2 / 29.8	L
1furA - 1jswA	413 / 1.749739	452 / 2.4 / 44.2	L
1furA - 1hylA	64 / 2.901541	- / - / -	G
1furA - 1i0aA	333 / 2.017358	389 / 3.3 / 29.8	L
1auwA - 1jswA	297 / 2.402125	383 / 3.3 / 29.9	L
1auwA - 1hylA	52 / 2.876209	- / - / -	G
1auwA - 1i0aA	445 / 0.681138	447 / 0.8 / 51.2	L
1jswA - 1hylA	59 / 2.819941	- / - / -	G
1jswA - 1i0aA	314 / 2.260573	386 / 3.2 / 30.1	L
1hylA - 1i0aA	49 / 2.787425	- / - / -	G
**1.25.30**			
1b3uA - 1bk6A	197 / 2.649229	155 / 4.7 / 11.5	G
1b3uA - 1gcjA	249 / 2.731531	340 / 5.6 / 17.9	L
1b3uA - 1ialA	202 / 2.696890	113 / 3.2 / 12.4	G
1b3uA - 1ibrA	67 / 2.743584	47 / 2.8 / 0.7	G
1b3uA - 1qbkB	225 / 2.930389	106 / 3.2 / 10.9	G
1b3uA - 2bct	177 / 2.817308	114 / 3.7 / 12.0	G
1bk6A - 1gcjA	245 / 2.452586	346 / 8.7 / 17.4	L
1bk6A - 1ialA	396 / 1.654236	412 / 1.9 / 46.5	L
1bk6A - 1ibrA	59 / 2.883340	40 / 3.3 / 0.1	G
1bk6A - 1qbkB	235 / 2.739448	328 / 7.3 / 15.1	L
1bk6A - 2bct	288 / 2.368801	398 / 4.0 / 29.9	L
1gcjA - 1ialA	254 / 2.535090	358 / 7.5 / 16.8	L
1gcjA - 1ibrA	67 / 2.792291	52 / 4.9 / 0.3	G
1gcjA - 1qbkB	310 / 2.385825	252 / 3.2 / 15.5	G

1gcjA - 2bct	226 / 2.833470	370 / 10.8 / 14.9	L
1ialA - 1ibrA	46 / 2.691598	48 / 3.2 / 0.4	L
1ialA - 1qbkB	227 / 2.665372	257 / 5.0 / 14.7	L
1ialA - 2bct	269 / 2.223326	403 / 5.3 / 28.4	L
1ibrA - 1qbkB	66 / 2.750472	- / - / -	G
1ibrA - 2bct	69 / 2.781739	39 / 3.0 /0.4	G
1qbkB - 2bct	231 / 2.661437	385 / 17.7 / 14.3	L
**2.30.110**			
1ci0A - 1dnlA	191 / 1.440915	195 / 1.6 / 24.0	L
1ci0A - 1ejeA	90 / 1.893767	97 / 4.2 / 5.9	L
1ci0A - 1i0rA	78 / 1.867700	89 / 2.5 / 6.6	L
1dnlA - 1ejeA	82 / 1.700216	91 / 3.2 / 5.9	L
1dnlA - 1i0rA	75 / 1.716565	88 /2.8 / 6.0	L
1ejeA - 1i0rA	139 / 1.617463	150 / 2.2 / 18.5	L
**2.40.100**			
1a33 - 1cynA	165 / 0.831118	166 / 0.9 / 30.6	L
1a33 - 1dywA	167 / 0.766534	172 / 1.2 / 32.5	L
1a33 - 1ihgA	173 / 0.764321	173 / 0.8 / 32.4	L
1a33 - 1lopA	140 / 1.139730	146 / 1.7 / 21.7	L
1a33 - 1qngA	166 / 0.917395	170 / 1.3 / 31.5	L
1a33 - 1qoiA	168 / 1.049905	169 / 1.2 / 30.5	L
1a33 - 2rmcA	165 / 0.811267	166 / 0.9 / 30.4	L
1cynA - 1dywA	163 / 0.873024	165 / 1.2 / 30.2	L
1cynA - 1ihgA	167 / 0.812465	169 / 1.2 / 30.1	L
1cynA - 1lopA	144 / 1.264397	147 / 1.7 / 22.0	L

1cynA - 1qngA	162 / 0.807030	164 / 1.2 / 30.1	L
1cynA - 1qoiA	164 / 0.711896	164 / 0.8 / 30.1	L
1cynA - 2rmcA	176 / 0.489924	178 / 0.8 / 34.9	L
1dywA - 1ihgA	170 / 0.784628	171 / 1.0 / 32.0	L
1dywA - 1lopA	141 / 1.354386	144 / 1.7 / 20.7	L
1dywA - 1qngA	168 / 0.640550	170 / 0.9 / 33.7	L
1dywA - 1qoiA	164 / 1.033299	168 / 1.5 / 30.0	L
1dywA - 2rmcA	159 / 0.634684	165 / 1.3 / 29.5	L
1ihgA - 1lopA	143 / 1.271632	145 / 1.6 / 20.8	L
1ihgA - 1qngA	167 / 0.840740	170 / 1.2 / 31.4	L
1ihgA - 1qoiA	165 / 0.755089	168 / 1.2 / 30.2	L
1ihgA - 2rmcA	166 / 0.842582	169 / 1.3 / 29.2	L
1lopA - 1qngA	141 / 1.528718	145 / 1.9 / 21.4	L
1lopA - 1qoiA	140 / 1.102437	146 / 1.8 / 21.5	L
1lopA - 2rmcA	143 / 1.216579	147 / 1.7 / 21.5	L
1qngA - 1qoiA	166 / 0.931126	169 / 1.3 / 31.3	L
1qngA - 2rmcA	162 / 0.929837	164 / 1.3 / 29.3	L
1qoiA - 2rmcA	165 / 0.870222	168 / 1.8 / 29.4	L
**2.100.10**			
1c3kA - 1ciy	118 / 2.517169	37 / 3.8 / 1.0	G
1c3kA - 1jacA	125 / 1.447366	55 / 4.3 / 0.5	G
1c3kA - 1jotA	127 / 1.528057	53 / 4.2 / 0.3	G
1c3kA - 1dlc	125 / 2.492074	41 / 3.2 / 1.4	G
1c3kA - 1vmoA	120 / 2.312039	53 / 3.7 / 0.5	G
1ciy - 1jacA	109 / 2.464506	115 / 2.5 / 9.4	L

1ciy - 1jotA	109 / 2.529496	114 / 2.3 / 9.4	B
1ciy - 1dlc	539 / 1.589934	558 / 2.0 / 44.1	L
1ciy - 1vmoA	125 / 2.589238	128 / 3.7 / 7.9	L
1jacA - 1jotA	133 / 0.360512	133 / 0.4 / 27.3	L
1jacA - 1dlc	115 / 2.684532	120 / 2.5 / 10.3	B
1jacA - 1vmoA	105 / 2.472352	122 / 3.1 / 11.7	L
1jotA - 1dlc	112 / 2.607978	120 / 2.5 / 10.3	B
1jotA - 1vmoA	101 / 2.366930	122 / 3.1 / 11.7	L
1dlc - 1vmoA	121 / 2.811144	137 / 3.4 / 9.2	L
**3.10.70**			
1bkf - 1grj	53 / 2.129308	56 / 2.4 / 4.9	L
1bkf - 1pbk	106 / 1.031962	107 / 1.3 / 19.1	L
1bkf - 1rot	105 / 1.586925	107 / 1.8 / 16.6	L
1bkf - 1yat	107 / 0.740635	107 / 0.8 / 20.5	L
1grj - 1pbk	48 / 2.230752	57 / 2.5 / 4.5	L
1grj - 1rot	49 / 2.533945	58 / 2.5 / 4.1	L
1grj - 1yat	53 / 2.097045	55 / 2.3 / 4.5	L
1pbk - 1rot	103 / 1.447314	108 / 1.8 / 15.9	L
1pbk - 1yat	107 / 0.956091	107 / 1.0 / 19.4	L
1rot - 1yat	111 / 1.526901	113 / 1.7 / 17.7	L
**3.40.91**			
1bhmA - 1cfr	92 / 2.541242	119 /4.5 / 4.5	L
1bhmA - 1d2iA	146 / 1.851088	149 / 2.7 / 15.6	L
1bhmA - 1fokA	84 / 2.652422	104 / 4.7 / 4.1	L
1cfr - 1d2iA	88 / 2.182160	103 / 3.6 / 4.6	L

1cfr - 1fokA	106 / 2.600421	136 / 4.2 / 5.7	L
1d2iA - 1fokA	91 / 2.755003	99 / 3.6 / 4.5	L
**3.70.10**			
1axcA - 1b77A	168 / 2.429400	202 / 3.4 / 15.6	L
1axcA - 1czdA	174 / 2.324310	205 / 3.5 / 16.1	L
1axcA - 1dmlA	181 / 2.777907	226 / 4.1 / 16.3	L
1axcA - 1ge8A	229 / 1.471087	236 / 1.8 / 30.0	L
1axcA - 1plq	247 / 1.327445	248 / 1.4 / 33.8	L
1b77A - 1czdA	228 / 0.600158	228 / 0.6 / 34.5	L
1b77A - 1dmlA	147 / 2.871160	110 / 2.8 / 10.7	L
1b77A - 1ge8A	169 / 2.436944	193 / 3.1 / 15.7	L
1b77A - 1plq	173 / 2.353772	200 / 3.1 / 15.6	L
1czdA - 1dmlA	152 / 2.828240	110 / 2.7 / 10.5	L
1czdA - 1ge8A	178 / 2.466398	196 / 3.2 / 15.6	L
1czdA - 1plq	177 / 2.344821	204 / 3.3 / 15.6	L
1dmlA - 1ge8A	165 / 2.723948	218 / 4.3 / 15.3	L
1dmlA - 1plq	177 / 2.827262	230 / 4.1 / 15.9	L
1ge8A - 1plq	222 / 1.595009	237 / 2.0 / 28.2	L
**2.40.20**			
1b2iA - 1ceaA	71 / 1.624921	79 / 1.6 / 12.9	B
1b2iA - 1kdu	68 / 2.131224	78 / 3.0 / 7.0	L
1b2iA - 1kiv	76 / 1.632192	78 / 1.8 / 12.4	L
1b2iA - 1krn	77 / 1.721116	79 / 1.9 / 12.6	L
1b2iA - 1pk4	75 / 1.711139	79 / 1.9 / 12.6	L
1b2iA - 1pmlA	75 / 1.991277	80 / 2.3 / 10.5	L

1ceaA - 1kdu	69 / 2.235091	74 / 2.2 / 8.2	B
1ceaA - 1kiv	77 / 0.924612	78 / 1.0 / 15.8	L
1ceaA - 1krn	78 / 0.935780	79 / 1.0 / 16.2	L
1ceaA - 1pk4	78 / 0.915780	79 / 1.0 / 16.3	L
1ceaA - 1pmlA	74 / 1.504394	77 / 1.5 / 12.6	L
1kdu - 1kiv	71 / 2.130127	77 / 2.5 / 8.1	L
1kdu - 1krn	63 / 1.793541	77 / 2.5 / 8.0	L
1kdu - 1pk4	68 / 2.072544	77 / 2.5 / 8.0	L
1kdu - 1pmlA	72 / 1.815999	84 / 2.5 / 10.1	L
1kiv - 1krn	78 / 0.637152	78 / 0.6 / 17.1	L
1kiv - 1pk4	78 / 0.595011	78 / 0.6 / 17.2	L
1kiv - 1pmlA	73 / 1.357903	76 / 1.5 / 13.1	L
1krn - 1pk4	79 / 0.152950	79 / 0.2 / 19.4	L
1krn - 1pmlA	73 / 1.826699	77 / 1.6 / 13.4	B
1pk4 - 1pmlA	73 / 1.425936	77 / 1.5 / 13.4	L

Structure Neighborhood based program:

PDBid1 - PDBid2	Matchprot2 (Seq. Nbhd.) (Lali / RMSD / Zscore)	DALI (Lali / RMSD / Zscore)	Good / Bad / Level
**1.10.64**			
1aq6A - 1c3uA	66 / 2.457820	60 / 3.6 / 1.0	G
1aq6A - 1fezA	168 / 2.175743	201 / 4.0 / 16.2	L
1aq6A - 1jud	216 / 1.232942	218 / 1.3 / 30.5	L
1aq6A - 1zrn	216 / 1.232157	218 / 1.3 / 30.9	L
1c3uA - 1fezA	76 / 2.204634	56 / 3.8 / 2.4	G
1c3uA - 1jud	7 / 0.195000	58 / 3.3 / 3.6	L
1c3uA - 1zrn	8 / 0.285602	59 / 3.1 / 3.3	L
1fezA - 1jud	157 / 2.020545	188 / 3.4 / 17.3	L
1fezA - 1zrn	159 / 2.043136	189 / 3.4 / 17.0	L
1jud - 1zrn	220 / 0.265135	220 / 0.3 / 39.1	L
**1.10.40**			
1rlr - 1yfm	79 / 2.457731	49 / 3.6 / 2.2	G
1rlr - 1furA	83 / 2.263151	48 / 3.7 / 2.3	G
1rlr - 1auwA	88 / 2.666284	60 / 3.3 / 3.1	G
1rlr - 1jswA	81 / 2.525280	63 / 4.6 / 1.2	G
1rlr - 1hylA	11 / 1.016705	- / - / -	L
1rlr - 1i0aA	77 / 2.455953	64 / 3.4 / 2.9	G
1yfm - 1furA	446 / 1.007248	446 / 1.0 / 50.4	L
1yfm - 1auwA	344 / 1.961941	389 / 2.9 / 31.4	L
1yfm - 1jswA	423 / 1.738966	443 / 1.9 / 48.9	L
1yfm - 1hylA	7 / 0.704219	- / - / -	L
1yfm - 1i0aA	344 / 2.157840	389 / 2.9 / 31.7	L

1furA - 1auwA	338 / 2.018078	386 / 3.2 / 29.8	L
1furA - 1jswA	413 / 1.812258	452 / 2.4 / 44.2	L
1furA - 1hylA	11 / 0.743377	- / - / -	L
1furA - 1i0aA	350 / 2.094681	389 / 3.3 / 29.8	L
1auwA - 1jswA	322 / 2.182510	383 / 3.3 / 29.9	L
1auwA - 1hylA	7 / 0.550223	- / - / -	L
1auwA - 1i0aA	445 / 0.660635	447 / 0.8 / 51.2	L
1jswA - 1hylA	18 / 2.069607	- / - / -	L
1jswA - 1i0aA	330 / 2.235845	386 / 3.2 / 30.1	L
1hylA - 1i0aA	13 / 1.311338	- / - / -	L
**1.25.30**			
1b3uA - 1bk6A	203 / 2.759172	155 / 4.7 / 11.5	G
1b3uA - 1gcjA	223 / 2.573049	340 / 5.6 / 17.9	L
1b3uA - 1ialA	195 / 2.788175	113 / 3.2 / 12.4	G
1b3uA - 1ibrA	16 / 1.197663	47 / 2.8 / 0.7	L
1b3uA - 1qbkB	202 / 2.707436	106 / 3.2 / 10.9	G
1b3uA - 2bct	176 / 2.616014	114 / 3.7 / 12.0	G
1bk6A - 1gcjA	249 / 2.446925	346 / 8.7 / 17.4	L
1bk6A - 1ialA	394 / 1.629739	412 / 1.9 / 46.5	L
1bk6A - 1ibrA	17 / 1.657662	40 / 3.3 / 0.1	L
1bk6A - 1qbkB	244 / 2.571325	328 / 7.3 / 15.1	L
1bk6A - 2bct	308 / 2.553301	398 / 4.0 / 29.9	L
1gcjA - 1ialA	244 / 2.565092	358 / 7.5 / 16.8	L
1gcjA - 1ibrA	43 / 2.214131	52 / 4.9 / 0.3	L
1gcjA - 1qbkB	260 / 2.651475	252 / 3.2 / 15.5	G

1gcjA - 2bct	231 / 2.426738	370 / 10.8 / 14.9	L
1ialA - 1ibrA	15 / 0.825930	48 / 3.2 / 0.4	L
1ialA - 1qbkB	254 / 2.724410	257 / 5.0 / 14.7	L
1ialA - 2bct	286 / 2.342282	403 / 5.3 / 28.4	L
1ibrA - 1qbkB	33 / 1.910666	- / - / -	L
1ibrA - 2bct	8 / 0.373141	39 / 3.0 /0.4	L
1qbkB - 2bct	224 / 2.603785	385 / 17.7 / 14.3	L
**2.30.110**			
1ci0A - 1dnlA	191 / 1.465403	195 / 1.6 / 24.0	L
1ci0A - 1ejeA	83 / 1.754242	97 / 4.2 / 5.9	L
1ci0A - 1i0rA	83 / 1.909922	89 / 2.5 / 6.6	L
1dnlA - 1ejeA	81 / 1.618309	91 / 3.2 / 5.9	L
1dnlA - 1i0rA	76 / 1.686808	88 /2.8 / 6.0	L
1ejeA - 1i0rA	144 / 1.667200	150 / 2.2 / 18.5	L
**2.40.100**			
1a33 - 1cynA	164 / 0.729849	166 / 0.9 / 30.6	L
1a33 - 1dywA	170 / 0.939079	172 / 1.2 / 32.5	L
1a33 - 1ihgA	173 / 0.764321	173 / 0.8 / 32.4	L
1a33 - 1lopA	142 / 1.153245	146 / 1.7 / 21.7	L
1a33 - 1qngA	167 / 0.997673	170 / 1.3 / 31.5	L
1a33 - 1qoiA	167 / 1.169084	169 / 1.2 / 30.5	L
1a33 - 2rmcA	165 / 0.778459	166 / 0.9 / 30.4	L
1cynA - 1dywA	161 / 0.728774	165 / 1.2 / 30.2	L
1cynA - 1ihgA	167 / 0.807851	169 / 1.2 / 30.1	L
1cynA - 1lopA	143 / 1.204773	147 / 1.7 / 22.0	L

1cynA - 1qngA	163 / 0.878122	164 / 1.2 / 30.1	L
1cynA - 1qoiA	165 / 0.765248	164 / 0.8 / 30.1	L
1cynA - 2rmcA	176 / 0.489924	178 / 0.8 / 34.9	L
1dywA - 1ihgA	169 / 0.681296	171 / 1.0 / 32.0	L
1dywA - 1lopA	141 / 1.371839	144 / 1.7 / 20.7	L
1dywA - 1qngA	169 / 0.707653	170 / 0.9 / 33.7	L
1dywA - 1qoiA	164 / 1.019824	168 / 1.5 / 30.0	L
1dywA - 2rmcA	161 / 0.784063	165 / 1.3 / 29.5	L
1ihgA - 1lopA	142 / 1.200484	145 / 1.6 / 20.8	L
1ihgA - 1qngA	167 / 0.826290	170 / 1.2 / 31.4	L
1ihgA - 1qoiA	165 / 0.755089	168 / 1.2 / 30.2	L
1ihgA - 2rmcA	166 / 0.832548	169 / 1.3 / 29.2	L
1lopA - 1qngA	139 / 1.283402	145 / 1.9 / 21.4	L
1lopA - 1qoiA	142 / 1.236044	146 / 1.8 / 21.5	L
1lopA - 2rmcA	143 / 1.175040	147 / 1.7 / 21.5	L
1qngA - 1qoiA	166 / 0.934620	169 / 1.3 / 31.3	L
1qngA - 2rmcA	163 / 0.995364	164 / 1.3 / 29.3	L
1qoiA - 2rmcA	165 / 0.926091	168 / 1.8 / 29.4	L
**2.100.10**			
1c3kA - 1ciy	120 / 2.110024	37 / 3.8 / 1.0	G
1c3kA - 1jacA	125 / 1.435965	55 / 4.3 / 0.5	G
1c3kA - 1jotA	127 / 1.523956	53 / 4.2 / 0.3	G
1c3kA - 1dlc	124 / 2.337422	41 / 3.2 / 1.4	G
1c3kA - 1vmoA	126 / 2.564556	53 / 3.7 / 0.5	G
1ciy - 1jacA	111 / 2.090545	115 / 2.5 / 9.4	L

1ciy - 1jotA	111 / 2.210455	114 / 2.3 / 9.4	L
1ciy - 1dlc	541 / 1.566309	558 / 2.0 / 44.1	L
1ciy - 1vmoA	122 / 2.623253	128 / 3.7 / 7.9	L
1jacA - 1jotA	133 / 0.360512	133 / 0.4 / 27.3	L
1jacA - 1dlc	113 / 2.107684	120 / 2.5 / 10.3	L
1jacA - 1vmoA	100 / 2.325070	122 / 3.1 / 11.7	L
1jotA - 1dlc	116 / 2.186783	120 / 2.5 / 10.3	L
1jotA - 1vmoA	110 / 2.533567	122 / 3.1 / 11.7	L
1dlc - 1vmoA	129 / 2.618661	137 / 3.4 / 9.2	L
**3.10.70**			
1bkf - 1grj	53 / 1.567967	56 / 2.4 / 4.9	L
1bkf - 1pbk	106 / 1.031962	107 / 1.3 / 19.1	L
1bkf - 1rot	104 / 1.471554	107 / 1.8 / 16.6	L
1bkf - 1yat	107 / 0.740635	107 / 0.8 / 20.5	L
1grj - 1pbk	51 / 1.281313	57 / 2.5 / 4.5	L
1grj - 1rot	47 / 1.641380	58 / 2.5 / 4.1	L
1grj - 1yat	53 / 1.512229	55 / 2.3 / 4.5	L
1pbk - 1rot	103 / 1.382043	108 / 1.8 / 15.9	L
1pbk - 1yat	107 / 0.957710	107 / 1.0 / 19.4	L
1rot - 1yat	109 / 1.433523	113 / 1.7 / 17.7	L
**3.40.91**			
1bhmA - 1cfr	91 / 2.177180	119 /4.5 / 4.5	L
1bhmA - 1d2iA	148 / 1.878168	149 / 2.7 / 15.6	L
1bhmA - 1fokA	63 / 2.727453	104 / 4.7 / 4.1	L
1cfr - 1d2iA	92 / 2.400749	103 / 3.6 / 4.6	L

1cfr - 1fokA	113 / 2.541903	136 / 4.2 / 5.7	L
1d2iA - 1fokA	80 / 2.488961	99 / 3.6 / 4.5	L
**3.70.10**			
1axcA - 1b77A	174 / 2.451725	202 / 3.4 / 15.6	L
1axcA - 1czdA	164 / 2.361052	205 / 3.5 / 16.1	L
1axcA - 1dmlA	183 / 2.760560	226 / 4.1 / 16.3	L
1axcA - 1ge8A	230 / 1.569787	236 / 1.8 / 30.0	L
1axcA - 1plq	247 / 1.380383	248 / 1.4 / 33.8	L
1b77A - 1czdA	228 / 0.600158	228 / 0.6 / 34.5	L
1b77A - 1dmlA	128 / 2.315777	110 / 2.8 / 10.7	G
1b77A - 1ge8A	171 / 2.654882	193 / 3.1 / 15.7	L
1b77A - 1plq	182 / 2.497389	200 / 3.1 / 15.6	L
1czdA - 1dmlA	131 / 2.422025	110 / 2.7 / 10.5	G
1czdA - 1ge8A	169 / 2.521506	196 / 3.2 / 15.6	L
1czdA - 1plq	167 / 2.215476	204 / 3.3 / 15.6	L
1dmlA - 1ge8A	158 / 2.745198	218 / 4.3 / 15.3	L
1dmlA - 1plq	175 / 2.704235	230 / 4.1 / 15.9	L
1ge8A - 1plq	221 / 1.455481	237 / 2.0 / 28.2	L
**2.40.20**			
1b2iA - 1ceaA	78 / 1.601455	79 / 1.6 / 12.9	L
1b2iA - 1kdu	68 / 2.102600	78 / 3.0 / 7.0	L
1b2iA - 1kiv	74 / 1.611512	78 / 1.8 / 12.4	L
1b2iA - 1krn	77 / 1.705257	79 / 1.9 / 12.6	L
1b2iA - 1pk4	78 / 1.796284	79 / 1.9 / 12.6	L
1b2iA - 1pmlA	75 / 1.959124	80 / 2.3 / 10.5	L

1ceaA - 1kdu	70 / 2.070622	74 / 2.2 / 8.2	L
1ceaA - 1kiv	77 / 0.905185	78 / 1.0 / 15.8	L
1ceaA - 1krn	78 / 0.935444	79 / 1.0 / 16.2	L
1ceaA - 1pk4	78 / 0.915780	79 / 1.0 / 16.3	L
1ceaA - 1pmlA	73 / 1.441332	77 / 1.5 / 12.6	L
1kdu - 1kiv	69 / 2.018335	77 / 2.5 / 8.1	L
1kdu - 1krn	70 / 1.982801	77 / 2.5 / 8.0	L
1kdu - 1pk4	69 / 2.016846	77 / 2.5 / 8.0	L
1kdu - 1pmlA	73 / 1.858452	84 / 2.5 / 10.1	L
1kiv - 1krn	78 / 0.637040	78 / 0.6 / 17.1	L
1kiv - 1pk4	78 / 0.595011	78 / 0.6 / 17.2	L
1kiv - 1pmlA	73 / 1.357903	76 / 1.5 / 13.1	L
1krn - 1pk4	79 / 0.152775	79 / 0.2 / 19.4	L
1krn - 1pmlA	75 / 1.520742	77 / 1.6 / 13.4	L
1pk4 - 1pmlA	74 / 1.424190	77 / 1.5 / 13.4	L

Comparison with CE

Fisher's Dataset

Sequence Neighborhood based Program

PDBid1-PDBid2	Matchprot2 (Lali / RMSD / Zscore)	CE (Lali / RMSD)	Good / Bad / Level
1dxtB - 1hbg	117 / 1.767981 / 10.883261	134 / 1.86	L
1cpcL - 1colA	89 / 2.345877 / 2.543957	116 / 3.25	L
1c2rA - 1ycc	94 / 1.824597 / 10.278438	98 / 1.94	L
2mtaC - 1ycc	74 / 1.907191 / 4.444056	76 / 2.02	L
1bbhA - 2ccyA	116 / 1.603838 / 13.049425	122 / 1.92	L
1bgeB - 2gmfA	85 / 2.480859 / 3.803303	102 / 4.02	L
1rcb - 2gmfA	76 / 2.211365 / 3.873671	105 / 4.42	L
1aep - 256bA	71 / 1.938330 / 3.276880	93 / 7.86	L
1osa - 4cpv	66 / 1.274414 / 5.304187	69 / 2.27	L
2sas - 2scpA	122 / 2.229376 / 7.007117	170 / 3.56	L
1hom - 1lfb	54 / 1.522779 / 5.542545	51 / 1.21	L
1lgaA - 2cyp	242 / 1.768907 / 15.013091	262 / 2.45	L
2hpdA - 2cpp	307 / 2.487783 / 8.767392	366 / 3.38	L
1fc1A - 2fb4H	109 / 1.894847 / 4.978134	137 / 3.45	L
2fbjL - 8fabB	180 / 1.810727 / 12.524636	194 / 2.19	L
1cid - 2rhe	91 / 2.059754 / 5.504575	98 / 2.97	L
1pfc - 3hlaB	80 / 2.259232 / 5.208956	97 / 3.44	L
1ten - 3hhrB	84 / 1.580910 / 7.230915	87 / 1.90	L
1tlk - 2rhe	87 / 1.580035 / 9.762585	88 / 1.93	L
3cd4 - 2rhe	88 / 1.381726 / 7.526783	92 / 1.95	L
3hlaB - 2rhe	70 / 2.269583 / 3.701798	85 / 3.46	L
1aaj - 1paz	80 / 1.564230 / 8.505390	80 / 1.78	G
2afnA - 1aozA	233 / 2.098379 / 6.903389	250 / 2.56	L

2azaA - 1paz	72 / 2.463617 / 2.403247	85 / 2.90	L
4sbvA - 2tbvA	155 / 1.482077 / 10.225784	157 / 1.90	L
1bbt1 - 2plv1	147 / 2.356716 / 4.801429	179 / 2.5	L
1sacA - 2ayh	127 / 2.592137 / 5.091943	148 / 3.72	L
1ltsD - 1bovA	63 / 2.317266 / 3.863577	68 / 2.34	L
1tie - 4fgf	105 / 2.209304 / 7.883892	115 / 2.86	L
8i1b - 4fgf	110 / 1.915536 / 11.460574	121 / 2.59	L
1arb - 5ptp	162 / 2.171203 / 10.283039	194 / 3.09	L
2sga - 5ptp	132 / 1.971517 / 10.002339	155 / 3.05	L
2snv - 5ptp	117 / 2.368816 / 5.778181	131 / 3.18	L
1mdc - 1ifc	127 / 2.512997 / 4.864672	128 / 1.92	L
1mup - 1rbp	121 / 2.120736 / 9.168372	143 / 2.96	L
2sim - 1nsbA	262 / 2.727954 / 8.422257	276 / 2.99	L
1cauB - 1cauA	153 / 2.057075 / 11.342371	160 / 2.02	B
2omf - 2por	240 / 2.259293 / 9.478756	266 / 3.04	L
1chrA - 2mnr	340 / 1.647549 / 20.115848	346 / 1.82	L
2mnr - 4enl	234 / 2.363370 / 6.154323	269 / 3.14	L
3rubL - 6xia	147 / 2.609368 / 0.911725	172 / 4.00	L
1crl - 1ede	160 / 2.503302 / 2.546601	220 / 3.91	L
1tahA - 1tca	172 / 2.020544 / 7.212261	188 / 2.39	L
1aba - 1ego	69 / 1.886953 / 6.671629	77 / 3.12	L
1dsbA - 2trxA	69 / 2.141120 / 2.644885	87 / 3.63	L
1gp1A - 2trxA	89 / 2.308867 / 5.505891	147 / 5.16	L
1atnA - 1atr	264 / 2.099867 / 10.675943	297 / 3.06	L
1hrhA - 1rnh	108 / 1.545015 / 11.875600	113 / 2.00	L

3chy - 2fox	91 / 2.370168 / 5.082222	109 / 3.68	L
2ak3A - 1gky	125 / 2.537671 / 5.006488	158 / 3.62	L
1gky - 3adk	124 / 2.093791 / 6.759818	154 / 2.94	L
2cmd - 6ldh	267 / 2.034366 / 15.927041	281 / 2.29	L
1eaf - 4cla	144 / 2.284240 / 6.764282	178 / 2.82	L
2gbp - 2liv	167 / 2.600682 / 3.336421	252 / 4.61	L
1mioC - 2minB	304 / 2.547204 / 4.974903	395 / 3.14	L
2pia - 1fnb	186 / 2.057759 / 8.453938	214 / 2.42	L
1gal - 3cox	372 / 2.337642 / 9.443108	415 / 3.20	L
1npx - 3grs	333 / 2.251019 / 9.270818	383 / 2.83	L
1fxiA - 1ubq	54 / 2.183049 / 2.676437	100 / 3.82	L
1cewI - 1molA	68 / 1.804976 / 5.625186	81 / 2.34	L
1stfI - 1molA	77 / 1.870025 / 7.557261	78 / 1.68	L
2pna - 1shaA	81 / 1.856416 / 7.029939	93 / 2.63	L
2sarA - 9rnt	61 / 2.485668 / 2.509794	84 / 4.48	L
1onc - 7rsa	90 / 2.201218 / 6.191860	98 / 2.24	L
5fd1 - 2fxb	52 / 1.684135 / 3.302135	65 / 3.55	L
2hhmA - 1fbpA	198 / 2.395615 / 8.102476	225 / 3.06	L
1hip - 2hipA	66 / 1.716334 / 9.245717	68 / 2.01	L
1isuA - 2hipA	54 / 1.692477 / 6.430629	54 / 1.90	G

Structure Neighborhood Based program

PDBid1 - PDBid2	Matchprot lali / rmsd / zscore	CE lali / rmsd	Good / Bad / Level
1dxtB - 1hbg	133 / 1.618052 / 15.527590	134 / 1.86	L
1cpcL - 1colA	91 / 2.453736 / 2.658678	116 / 3.25	L
1c2rA - 1ycc	95 / 1.362028 / 12.269984	98 / 1.94	L
2mtaC - 1ycc	78 / 1.941872 / 4.895345	76 / 2.02	G
1bbhA - 2ccyA	121 / 1.764434 / 13.536841	122 / 1.92	L
1bgeB - 2gmfA	87 / 2.225433 / 5.446246	102 / 4.02	L
1rcb - 2gmfA	77 / 1.879182 / 5.082635	105 / 4.42	L
1aep - 256bA	64 / 2.117783 / 1.639862	93 / 7.86	L
1osa - 4cpv	65 / 1.526141 / 4.545486	69 / 2.27	L
2sas - 2scpA	138 / 2.231157 / 9.750968	170 / 3.56	L
1hom - 1lfb	52 / 1.281308 / 5.652445	51 / 1.21	L
1lgaA - 2cyp	238 / 2.192340 / 12.818557	262 / 2.45	L
2hpdA - 2cpp	283 / 2.585693 / 6.934695	366 / 3.38	L
1fc1A - 2fb4H	112 / 1.892321 / 4.922228	137 / 3.45	L
2fbjL - 8fabB	185 / 1.735389 / 13.378943	194 / 2.19	L
1cid - 2rhe	91 / 2.068938 / 5.552895	98 / 2.97	L
1pfc - 3hlaB	85 / 2.230198 / 6.537908	97 / 3.44	L
1ten - 3hhrB	82 / 1.392378 / 7.169996	87 / 1.90	L
1tlk - 2rhe	84 / 1.347123 / 10.106946	88 / 1.93	L
3cd4 - 2rhe	88 / 1.596862 / 6.705303	92 / 1.95	L
3hlaB - 2rhe	68 / 2.266716 / 3.652379	85 / 3.46	L
1aaj - 1paz	80 / 1.488212 / 8.657101	80 / 1.78	G
2afnA - 1aozA	227 / 2.134964 / 5.970371	250 / 2.56	L

2azaA - 1paz	79 / 2.208511 / 4.535960	85 / 2.90	L
4sbvA - 2tbvA	154 / 1.573555 / 10.004015	157 / 1.90	L
1bbt1 - 2plv1	156 / 2.045998 / 7.154092	179 / 2.5	L
1sacA - 2ayh	139 / 2.453903 / 7.253736	148 / 3.72	L
1ltsD - 1bovA	67 / 1.769267 / 6.901099	68 / 2.34	L
1tie - 4fgf	106 / 2.279553 / 8.261742	115 / 2.86	L
8i1b - 4fgf	110 / 1.901800 / 11.803427	121 / 2.59	L
1arb - 5ptp	168 / 1.986419 / 11.874460	194 / 3.09	L
2sga - 5ptp	129 / 1.833044 / 10.346103	155 / 3.05	L
2snv - 5ptp	96 / 2.290828 / 3.292010	131 / 3.18	L
1mdc - 1ifc	- / - / -	128 / 1.92	L
1mup - 1rbp	122 / 2.009872 / 10.061081	143 / 2.96	L
2sim - 1nsbA	262 / 2.632144 / 8.488201	276 / 2.99	L
1cauB - 1cauA	152 / 1.738604 / 13.203211	160 / 2.02	L
2omf - 2por	235 / 2.467897 / 8.573208	266 / 3.04	L
1chrA - 2mnr	335 / 1.664985 / 19.321587	346 / 1.82	L
2mnr - 4enl	230 / 2.517675 / 5.566740	269 / 3.14	L
3rubL - 6xia	152 / 2.796273 / 0.795955	172 / 4.00	L
1crl - 1ede	174 / 2.491762 / 3.063029	220 / 3.91	L
1tahA - 1tca	168 / 2.416043 / 5.176979	188 / 2.39	L
1aba - 1ego	69 / 2.000439 / 6.547013	77 / 3.12	L
1dsbA - 2trxA	76 / 1.839218 / 4.229998	87 / 3.63	L
1gp1A - 2trxA	86 / 1.886982 / 5.712329	147 / 5.16	L
1atnA - 1atr	260 / 2.181434 / 10.128426	297 / 3.06	L
1hrhA - 1rnh	108 / 1.511972 / 12.136479	113 / 2.00	L

3chy - 2fox	91 / 2.430114 / 5.063224	109 / 3.68	L
2ak3A - 1gky	135 / 2.352513 / 6.312533	158 / 3.62	L
1gky - 3adk	141 / 2.253797 / 8.316162	154 / 2.94	L
2cmd - 6ldh	274 / 2.056520 / 16.639841	281 / 2.29	L
1eaf - 4cla	160 / 2.022917 / 10.536119	178 / 2.82	L
2gbp - 2liv	169 / 2.544403 / 4.131064	252 / 4.61	L
1mioC - 2minB	318 / 2.330087 / 5.902166	395 / 3.14	L
2pia - 1fnb	195 / 1.855691 / 9.533363	214 / 2.42	L
1gal - 3cox	366 / 2.119449 / 10.096328	415 / 3.20	L
1npx - 3grs	345 / 2.197732 / 10.535785	383 / 2.83	L
1fxiA - 1ubq	56 / 2.163154 / 3.071374	100 / 3.82	L
1cewI - 1molA	79 / 1.944987 / 7.305458	81 / 2.34	L
1stfI - 1molA	82 / 1.852846 / 9.165097	78 / 1.68	L
2pna - 1shaA	83 / 1.941611 / 7.092368	93 / 2.63	L
2sarA - 9rnt	64 / 2.298871 / 3.773092	84 / 4.48	L
1onc - 7rsa	93 / 1.296087 / 12.070139	98 / 2.24	L
5fd1 - 2fxb	55 / 1.879364 / 3.600283	65 / 3.55	L
2hhmA - 1fbpA	187 / 2.103918 / 8.559585	225 / 3.06	L
1hip - 2hipA	65 / 1.462879 / 8.980166	68 / 2.01	L
1isuA - 2hipA	53 / 1.497409 / 6.679889	54 / 1.90	L

Novotny's Dataset

Sequence Neighborhood based program

PDBid1 - PDBid2	Matchprot2 (Seq. Nbhd.) (Lali / RMSD / Zscore)	CE (Lali / RMSD)	Good / Bad / Level
**1.10.164**			
1aq6A - 1c3uA	52 / 2.552922 / -1.412559	96 / 4.73	L
1aq6A - 1fezA	161 / 1.993285 / 9.783233	202 / 3.53	L
1aq6A - 1jud	217 / 1.304811 / 22.383812	218 / 1.30	L
1aq6A - 1zrn	217 / 1.292294 / 22.690917	218 / 1.29	L
1c3uA - 1fezA	55 / 2.231143 / -1.211438	95 / 5.75	L
1c3uA - 1jud	15 / 0.953262 / -1.856251	102 / 4.88	L
1c3uA - 1zrn	78 / 2.630662 / -0.834005	101 / 4.74	L
1fezA - 1jud	157 / 1.974788 / 9.996566	194 / 3.37	L
1fezA - 1zrn	163 / 2.162118 / 10.277151	194 / 3.35	L
1jud - 1zrn	220 / 0.265135 / 30.214916	220 / 0.27	L
**1.10.40**			
1rlr - 1yfm	95 / 3.074015 / -1.563337	172 / 7.63	L
1rlr - 1furA	85 / 2.771679 / -1.603553	174 / 7.63	L
1rlr - 1auwA	85 / 2.734123 / -1.419069	187 / 7.68	L
1rlr - 1jswA	86 / 2.680286 / -1.513539	173 / 7.42	L
1rlr - 1hylA	62 / 2.972107 / -1.588022	134 / 6.25	L
1rlr - 1i0aA	79 / 2.859903 / -1.749958	187 / 7.69	L
1yfm - 1furA	446 / 1.007248 / 22.026687	446 / 1.01	L
1yfm - 1auwA	347 / 2.131289 / 9.574798	383 / 2.65	L
1yfm - 1jswA	423 / 1.807071 / 16.095213	443 / 1.85	L
1yfm - 1hylA	55 / 2.680767 / -1.400364	119 / 6.10	L
1yfm - 1i0aA	350 / 1.964233 / 10.218229	391 / 2.84	L

1furA - 1auwA	342 / 1.942800 / 10.100266	381 / 2.77	L
1furA - 1jswA	413 / 1.749739 / 16.810692	452 / 2.38	L
1furA - 1hylA	64 / 2.901541 / -1.251467	118 / 7.42	L
1furA - 1i0aA	333 / 2.017358 / 9.289516	388 / 2.90	L
1auwA - 1jswA	297 / 2.402125 / 6.362020	365 / 2.70	L
1auwA - 1hylA	52 / 2.876209 / -1.581114	118 / 5.59	L
1auwA - 1i0aA	445 / 0.681138 / 21.504745	446 / 0.71	L
1jswA - 1hylA	59 / 2.819941 / -1.492591	126 / 5.35	L
1jswA - 1i0aA	314 / 2.260573 / 7.221459	387 / 3.02	L
1hylA - 1i0aA	49 / 2.787425 / -1.673912	118 / 5.61	L
**1.25.30**			
1b3uA - 1bk6A	197 / 2.649229 / 1.077622	293 / 5.05	L
1b3uA - 1gcjA	249 / 2.731531 / 2.003215	377 / 4.72	L
1b3uA - 1ialA	202 / 2.696890 / 1.073810	278 / 4.66	L
1b3uA - 1ibrA	67 / 2.743584 / -0.973410	108 / 4.96	L
1b3uA - 1qbkB	225 / 2.930389 / -0.880217	343 / 4.71	L
1b3uA - 2bct	177 / 2.817308 / -0.072292	308 / 5.66	L
1bk6A - 1gcjA	245 / 2.452586 / 3.966035	342 / 4.04	L
1bk6A - 1ialA	396 / 1.654236 / 18.426181	413 / 1.93	L
1bk6A - 1ibrA	59 / 2.883340 / -1.146076	107 / 6.55	L
1bk6A - 1qbkB	235 / 2.739448 / 0.602050	303 / 3.83	L
1bk6A - 2bct	288 / 2.368801 / 6.058353	385 / 3.22	L
1gcjA - 1ialA	254 / 2.535090 / 4.109992	310 / 4.09	L
1gcjA - 1ibrA	67 / 2.792291 / -1.024028	120 / 6.14	L
1gcjA - 1qbkB	310 / 2.385825 / 2.201474	304 / 3.69	G

1gcjA - 2bct	226 / 2.833470 / 1.579869	322 / 4.32	L
1ialA - 1ibrA	46 / 2.691598 / -1.501617	149 / 5.86	L
1ialA - 1qbkB	227 / 2.665372 / 0.732473	323 / 4.27	L
1ialA - 2bct	269 / 2.223326 / 5.451147	384 / 3.96	L
1ibrA - 1qbkB	66 / 2.750472 / -1.307502	75 / 4.99	L
1ibrA - 2bct	69 / 2.781739 / -0.963062	108 / 6.72	L
1qbkB - 2bct	231 / 2.661437 / 0.222369	320 / 4.66	L
**2.30.110**			
1ci0A - 1dnlA	191 / 1.440915 / 18.765724	198 / 1.58	L
1ci0A - 1ejeA	90 / 1.893767 / 4.037346	98 / 6.80	L
1ci0A - 1i0rA	78 / 1.867700 / 3.360269	117 / 4.31	L
1dnlA - 1ejeA	82 / 1.700216 / 3.516565	99 / 7.12	L
1dnlA - 1i0rA	75 / 1.716565 / 2.999491	93 / 3.10	L
1ejeA - 1i0rA	139 / 1.617463 / 14.237837	147 / 2.03	L
**2.40.100**			
1a33 - 1cynA	165 / 0.831118 / 25.581584	164 / 0.73	L
1a33 - 1dywA	167 / 0.766534 / 27.063242	171 / 1.07	L
1a33 - 1ihgA	173 / 0.764321 / 17.459092	172 / 0.71	L
1a33 - 1lopA	140 / 1.139730 / 17.874507	145 / 1.47	L
1a33 - 1qngA	166 / 0.917395 / 26.507498	169 / 1.23	L
1a33 - 1qoiA	168 / 1.049905 / 25.745149	168 / 1.24	L
1a33 - 2rmcA	165 / 0.811267 / 25.329352	164 / 0.72	L
1cynA - 1dywA	163 / 0.873024 / 25.377609	163 / 0.94	G
1cynA - 1ihgA	167 / 0.812465 / 16.103729	164 / 0.65	L
1cynA - 1lopA	144 / 1.264397 / 18.516408	145 / 1.38	L

1cynA - 1qngA	162 / 0.807030 / 25.441336	162 / 0.94	G
1cynA - 1qoiA	164 / 0.711896 / 25.489766	163 / 0.70	L
1cynA - 2rmcA	176 / 0.489924 / 28.988623	176 / 0.55	G
1dywA - 1ihgA	170 / 0.784628 / 17.359629	170 / 0.93	G
1dywA - 1lopA	141 / 1.354386 / 17.440849	144 / 1.68	L
1dywA - 1qngA	168 / 0.640550 / 28.546519	169 / 0.71	L
1dywA - 1qoiA	164 / 1.033299 / 25.202564	164 / 1.13	G
1dywA - 2rmcA	159 / 0.634684 / 24.415682	163 / 1.04	L
1ihgA - 1lopA	143 / 1.271632 / 11.138583	147 / 1.67	L
1ihgA - 1qngA	167 / 0.840740 / 16.883894	169 / 1.12	L
1ihgA - 1qoiA	165 / 0.755089 / 16.185676	165 / 0.76	L
1ihgA - 2rmcA	166 / 0.842582 / 15.402875	164 / 0.75	L
1lopA - 1qngA	141 / 1.528718 / 17.161924	143 / 1.71	L
1lopA - 1qoiA	140 / 1.102437 / 18.060041	144 / 1.49	L
1lopA - 2rmcA	143 / 1.216579 / 17.977920	145 / 1.38	L
1qngA - 1qoiA	166 / 0.931126 / 26.442762	167 / 1.17	L
1qngA - 2rmcA	162 / 0.929837 / 24.657075	162 / 1.06	G
1qoiA - 2rmcA	165 / 0.870222 / 24.725030	163 / 0.77	L
**2.100.10**			
1c3kA - 1ciy	118 / 2.517169 / 2.564285	118 / 2.64	G
1c3kA - 1jacA	125 / 1.447366 / 15.925442	120 / 1.53	G
1c3kA - 1jotA	127 / 1.528057 / 16.300306	120 / 1.53	G
1c3kA - 1dlc	125 / 2.492074 / 2.989941	120 / 2.56	G
1c3kA - 1vmoA	120 / 2.312039 / 9.734756	133 / 2.77	L
1ciy - 1jacA	109 / 2.464506 / 2.133106	117 / 3.13	L

1ciy - 1jotA	109 / 2.529496 / 2.042840	120 / 2.83	L
1ciy - 1dlc	539 / 1.589934 / 17.736200	560 / 1.92	L
1ciy - 1vmoA	125 / 2.589238 / 2.103541	145 / 3.83	L
1jacA - 1jotA	133 / 0.360512 / 24.617682	133 / 0.36	L
1jacA - 1dlc	115 / 2.684532 / 1.764475	127 / 3.45	L
1jacA - 1vmoA	105 / 2.472352 / 6.645189	119 / 2.94	L
1jotA - 1dlc	112 / 2.607978 / 1.779138	127 / 3.44	L
1jotA - 1vmoA	101 / 2.366930 / 6.039785	119 / 2.91	L
1dlc - 1vmoA	121 / 2.811144 / 1.283490	143 / 3.48	L
**3.10.70**			
1bkf - 1grj	53 / 2.129308 / 1.518653	109 / 2.52	L
1bkf - 1pbk	106 / 1.031962 / 17.559391	107 / 1.27	L
1bkf - 1rot	105 / 1.586925 / 14.708936	107 / 1.75	L
1bkf - 1yat	107 / 0.740635 / 18.854510	106 / 0.70	L
1grj - 1pbk	48 / 2.230752 / 0.214513	117 / 3.03	L
1grj - 1rot	49 / 2.533945 / -0.305146	111 / 2.84	L
1grj - 1yat	53 / 2.097045 / 1.407873	115 / 3.26	L
1pbk - 1rot	103 / 1.447314 / 13.590910	108 / 1.80	L
1pbk - 1yat	107 / 0.956091 / 17.711989	108 / 1.23	L
1rot - 1yat	111 / 1.526901 / 15.852777	108 / 1.59	G
**3.40.91**			
1bhmA - 1cfr	92 / 2.541242 / 1.558147	139 / 3.99	L
1bhmA - 1d2iA	146 / 1.851088 / 10.514207	171 / 3.29	L
1bhmA - 1fokA	84 / 2.652422 / -0.426451	103 / 3.80	L
1cfr - 1d2iA	88 / 2.182160 / 1.681056	129 / 5.87	L

1cfr - 1fokA	106 / 2.600421 / 0.036905	169 / 5.77	L
1d2iA - 1fokA	91 / 2.755003 / -0.468240	102 / 3.68	L
**3.70.10**			
1axcA - 1b77A	168 / 2.429400 / 8.210375	201 / 3.33	L
1axcA - 1czdA	174 / 2.324310 / 9.465431	200 / 3.25	L
1axcA - 1dmlA	181 / 2.777907 / 6.577876	223 / 3.71	L
1axcA - 1ge8A	229 / 1.471087 / 21.067522	234 / 1.79	L
1axcA - 1plq	247 / 1.327445 / 23.877194	248 / 1.37	L
1b77A - 1czdA	228 / 0.600158 / 26.084288	228 / 0.60	L
1b77A - 1dmlA	147 / 2.871160 / 3.251943	208 / 4.18	L
1b77A - 1ge8A	169 / 2.436944 / 8.313997	197 / 3.14	L
1b77A - 1plq	173 / 2.353772 / 9.372677	211 / 3.59	L
1czdA - 1dmlA	152 / 2.828240 / 3.726109	205 / 4.10	L
1czdA - 1ge8A	178 / 2.466398 / 9.430839	199 / 3.21	L
1czdA - 1plq	177 / 2.344821 / 9.793609	194 / 3.43	L
1dmlA - 1ge8A	165 / 2.723948 / 4.926716	220 / 4.06	L
1dmlA - 1plq	177 / 2.827262 / 6.036022	225 / 3.66	L
1ge8A - 1plq	222 / 1.595009 / 18.242090	237 / 1.95	L
**2.40.20**			
1b2iA - 1ceaA	71 / 1.624921 / 9.262427	79 / 1.60	B
1b2iA - 1kdu	68 / 2.131224 / 5.320875	73 / 2.64	L
1b2iA - 1kiv	76 / 1.632192 / 11.183288	76 / 1.69	G
1b2iA - 1krn	77 / 1.721116 / 10.402196	71 / 1.63	L
1b2iA - 1pk4	75 / 1.711139 / 10.876151	71 / 1.62	L
1b2iA - 1pmlA	75 / 1.991277 / 8.618455	81 / 2.36	L

1ceaA - 1kdu	69 / 2.235091 / 5.622503	76 / 2.39	L
1ceaA - 1kiv	77 / 0.924612 / 14.632626	75 / 0.84	L
1ceaA - 1krn	78 / 0.935780 / 14.157968	78 / 0.94	L
1ceaA - 1pk4	78 / 0.915780 / 15.184383	78 / 0.92	L
1ceaA - 1pmlA	74 / 1.504394 / 10.624290	79 / 1.80	L
1kdu - 1kiv	71 / 2.130127 / 6.895939	76 / 2.35	L
1kdu - 1krn	63 / 1.793541 / 4.976156	77 / 2.48	L
1kdu - 1pk4	68 / 2.072544 / 6.073593	77 / 2.47	L
1kdu - 1pmlA	72 / 1.815999 / 8.075270	80 / 2.08	L
1kiv - 1krn	78 / 0.637152 / 15.177455	78 / 0.64	L
1kiv - 1pk4	78 / 0.595011 / 16.300816	78 / 0.60	L
1kiv - 1pmlA	73 / 1.357903 / 11.411978	78 / 1.78	L
1krn - 1pk4	79 / 0.152950 / 17.384359	79 / 0.15	L
1krn - 1pmlA	73 / 1.826699 / 8.854390	79 / 1.85	L
1pk4 - 1pmlA	73 / 1.425936 / 11.459319	79 / 1.87	L

Structure Neighborhood based program

PDBid1 - PDBid2	Matchprot2 (Struct. Nbhd.) (Lali / RMSD / Zscore)	CE (Lali / RMSD)	Good / Bad / Level
**1.10.64**			
1aq6A - 1c3uA	66 / 2.457820 / -0.975812	96 / 4.73	L
1aq6A - 1fezA	168 / 2.175743 / 10.014351	202 / 3.53	L
1aq6A - 1jud	216 / 1.232942 / 22.438126	218 / 1.30	L
1aq6A - 1zrn	216 / 1.232157 / 22.631065	218 / 1.29	L
1c3uA - 1fezA	76 / 2.204634 / -0.478927	95 / 5.75	L
1c3uA - 1jud	7 / 0.195000 / -1.954991	102 / 4.88	L
1c3uA - 1zrn	8 / 0.285602 / -1.941053	101 / 4.74	L
1fezA - 1jud	157 / 2.020545 / 9.813373	194 / 3.37	L
1fezA - 1zrn	159 / 2.043136 / 10.014177	194 / 3.35	L
1jud - 1zrn	220 / 0.265135 / 30.214916	220 / 0.27	L
**1.10.40**			
1rlr - 1yfm	79 / 2.457731 / -1.531181	172 / 7.63	L
1rlr - 1furA	83 / 2.263151 / -1.504402	174 / 7.63	L
1rlr - 1auwA	88 / 2.666284 / -1.397462	187 / 7.68	L
1rlr - 1jswA	81 / 2.525280 / -1.547428	173 / 7.42	L
1rlr - 1hylA	11 / 1.016705 / -1.940894	134 / 6.25	L
1rlr - 1i0aA	77 / 2.455953 / -1.621795	187 / 7.69	L
1yfm - 1furA	446 / 1.007248 / 22.026687	446 / 1.01	L
1yfm - 1auwA	344 / 1.961941 / 10.112582	383 / 2.65	L
1yfm - 1jswA	423 / 1.738966 / 16.686973	443 / 1.85	L
1yfm - 1hylA	7 / 0.704219 / -1.965680	119 / 6.10	L
1yfm - 1i0aA	344 / 2.157840 / 9.397772	391 / 2.84	L

1furA - 1auwA	338 / 2.018078 / 9.832389	381 / 2.77	L
1furA - 1jswA	413 / 1.812258 / 16.035052	452 / 2.38	L
1furA - 1hylA	11 / 0.743377 / -1.918575	118 / 7.42	L
1furA - 1i0aA	350 / 2.094681 / 10.328766	388 / 2.90	L
1auwA - 1jswA	322 / 2.182510 / 7.833997	365 / 2.70	L
1auwA - 1hylA	7 / 0.550223 / -1.962580	118 / 5.59	L
1auwA - 1i0aA	445 / 0.660635 / 21.532919	446 / 0.71	L
1jswA - 1hylA	18 / 2.069607 / -1.892759	126 / 5.35	L
1jswA - 1i0aA	330 / 2.235845 / 8.207263	387 / 3.02	L
1hylA - 1i0aA	13 / 1.311338 / -1.891736	118 / 5.61	L
**1.25.30**			
1b3uA - 1bk6A	203 / 2.759172 / 1.192922	293 / 5.05	L
1b3uA - 1gcjA	223 / 2.573049 / 1.412590	377 / 4.72	L
1b3uA - 1ialA	195 / 2.788175 / 0.715012	278 / 4.66	L
1b3uA - 1ibrA	16 / 1.197663 / -1.869836	108 / 4.96	L
1b3uA - 1qbkB	202 / 2.707436 / -0.849227	343 / 4.71	L
1b3uA - 2bct	176 / 2.616014 / 0.237968	308 / 5.66	L
1bk6A - 1gcjA	249 / 2.446925 / 4.374222	342 / 4.04	L
1bk6A - 1ialA	394 / 1.629739 / 18.051942	413 / 1.93	L
1bk6A - 1ibrA	17 / 1.657662 / -1.794449	107 / 6.55	L
1bk6A - 1qbkB	244 / 2.571325 / 1.146742	303 / 3.83	L
1bk6A - 2bct	308 / 2.553301 / 6.277557	385 / 3.22	L
1gcjA - 1ialA	244 / 2.565092 / 3.769208	310 / 4.09	L
1gcjA - 1ibrA	43 / 2.214131 / -1.274271	120 / 6.14	L
1gcjA - 1qbkB	260 / 2.651475 / 1.230772	304 / 3.69	L

1gcjA - 2bct	231 / 2.426738 / 2.757577	322 / 4.32	L
1ialA - 1ibrA	15 / 0.825930 / -1.798085	149 / 5.86	L
1ialA - 1qbkB	254 / 2.724410 / 0.985744	323 / 4.27	L
1ialA - 2bct	286 / 2.342282 / 5.748118	384 / 3.96	L
1ibrA - 1qbkB	33 / 1.910666 / -1.685608	75 / 4.99	L
1ibrA - 2bct	8 / 0.373141 / -1.940882	108 / 6.72	L
1qbkB - 2bct	224 / 2.603785 / 0.147578	320 / 4.66	L
**2.30.110**			
1ci0A - 1dnlA	191 / 1.465403 / 18.749262	198 / 1.58	L
1ci0A - 1ejeA	83 / 1.754242 / 3.534641	98 / 6.80	L
1ci0A - 1i0rA	83 / 1.909922 / 3.818560	117 / 4.31	L
1dnlA - 1ejeA	81 / 1.618309 / 3.399219	99 / 7.12	L
1dnlA - 1i0rA	76 / 1.686808 / 2.943237	93 / 3.10	L
1ejeA - 1i0rA	144 / 1.667200 / 14.952628	147 / 2.03	L
**2.40.100**			
1a33 - 1cynA	164 / 0.729849 / 25.560363	164 / 0.73	L
1a33 - 1dywA	170 / 0.939079 / 27.346710	171 / 1.07	L
1a33 - 1ihgA	173 / 0.764321 / 17.459092	172 / 0.71	L
1a33 - 1lopA	142 / 1.153245 / 18.416710	145 / 1.47	L
1a33 - 1qngA	167 / 0.997673 / 26.510461	169 / 1.23	L
1a33 - 1qoiA	167 / 1.169084 / 24.947909	168 / 1.24	L
1a33 - 2rmcA	165 / 0.778459 / 25.405342	164 / 0.72	L
1cynA - 1dywA	161 / 0.728774 / 25.253658	163 / 0.94	L
1cynA - 1ihgA	167 / 0.807851 / 16.038324	164 / 0.65	L
1cynA - 1lopA	143 / 1.204773 / 18.310080	145 / 1.38	L

1cynA - 1qngA	163 / 0.878122 / 25.478743	162 / 0.94	G
1cynA - 1qoiA	165 / 0.765248 / 25.616371	163 / 0.70	L
1cynA - 2rmcA	176 / 0.489924 / 28.988623	176 / 0.55	G
1dywA - 1ihgA	169 / 0.681296 / 17.367192	170 / 0.93	L
1dywA - 1lopA	141 / 1.371839 / 17.660921	144 / 1.68	L
1dywA - 1qngA	169 / 0.707653 / 28.690540	169 / 0.71	L
1dywA - 1qoiA	164 / 1.019824 / 25.218047	164 / 1.13	G
1dywA - 2rmcA	161 / 0.784063 / 24.592025	163 / 1.04	L
1ihgA - 1lopA	142 / 1.200484 / 11.116520	147 / 1.67	L
1ihgA - 1qngA	167 / 0.826290 / 16.990349	169 / 1.12	L
1ihgA - 1qoiA	165 / 0.755089 / 16.185676	165 / 0.76	L
1ihgA - 2rmcA	166 / 0.832548 / 15.404889	164 / 0.75	L
1lopA - 1qngA	139 / 1.283402 / 17.698926	143 / 1.71	L
1lopA - 1qoiA	142 / 1.236044 / 18.249012	144 / 1.49	L
1lopA - 2rmcA	143 / 1.175040 / 18.156528	145 / 1.38	L
1qngA - 1qoiA	166 / 0.934620 / 26.360490	167 / 1.17	L
1qngA - 2rmcA	163 / 0.995364 / 24.698286	162 / 1.06	G
1qoiA - 2rmcA	165 / 0.926091 / 24.565419	163 / 0.77	L
**2.100.10**			
1c3kA - 1ciy	120 / 2.110024 / 3.368612	118 / 2.64	G
1c3kA - 1jacA	125 / 1.435965 / 16.151930	120 / 1.53	G
1c3kA - 1jotA	127 / 1.523956 / 16.300536	120 / 1.53	G
1c3kA - 1dlc	124 / 2.337422 / 3.054529	120 / 2.56	G
1c3kA - 1vmoA	126 / 2.564556 / 9.449866	133 / 2.77	L
1ciy - 1jacA	111 / 2.090545 / 2.985861	117 / 3.13	L

1ciy - 1jotA	111 / 2.210455 / 2.679259	120 / 2.83	L
1ciy - 1dlc	541 / 1.566309 / 17.752113	560 / 1.92	L
1ciy - 1vmoA	122 / 2.623253 / 2.103426	145 / 3.83	L
1jacA - 1jotA	133 / 0.360512 / 24.617682	133 / 0.36	L
1jacA - 1dlc	113 / 2.107684 / 3.212410	127 / 3.45	L
1jacA - 1vmoA	100 / 2.325070 / 5.552021	119 / 2.94	L
1jotA - 1dlc	116 / 2.186783 / 3.209516	127 / 3.44	L
1jotA - 1vmoA	110 / 2.533567 / 8.273235	119 / 2.91	L
1dlc - 1vmoA	129 / 2.618661 / 2.558328	143 / 3.48	L
**3.10.70**			
1bkf - 1grj	53 / 1.567967 / 2.578350	109 / 2.52	L
1bkf - 1pbk	106 / 1.031962 / 17.559391	107 / 1.27	L
1bkf - 1rot	104 / 1.471554 / 14.743686	107 / 1.75	L
1bkf - 1yat	107 / 0.740635 / 18.854510	106 / 0.70	L
1grj - 1pbk	51 / 1.281313 / 2.236802	117 / 3.03	L
1grj - 1rot	47 / 1.641380 / 0.936361	111 / 2.84	L
1grj - 1yat	53 / 1.512229 / 2.352950	115 / 3.26	L
1pbk - 1rot	103 / 1.382043 / 13.859318	108 / 1.80	L
1pbk - 1yat	107 / 0.957710 / 17.727875	108 / 1.23	L
1rot - 1yat	109 / 1.433523 / 15.401467	108 / 1.59	G
**3.40.91**			
1bhmA - 1cfr	91 / 2.177180 / 2.025581	139 / 3.99	L
1bhmA - 1d2iA	148 / 1.878168 / 10.815694	171 / 3.29	L
1bhmA - 1fokA	63 / 2.727453 / -1.303968	103 / 3.80	L
1cfr - 1d2iA	92 / 2.400749 / 1.391207	129 / 5.87	L

1cfr - 1fokA	113 / 2.541903 / 0.407745	169 / 5.77	L
1d2iA - 1fokA	80 / 2.488961 / -0.469540	102 / 3.68	L
**3.70.10**			
1axcA - 1b77A	174 / 2.451725 / 8.847162	201 / 3.33	L
1axcA - 1czdA	164 / 2.361052 / 8.345812	200 / 3.25	L
1axcA - 1dmlA	183 / 2.760560 / 6.466431	223 / 3.71	L
1axcA - 1ge8A	230 / 1.569787 / 21.042094	234 / 1.79	L
1axcA - 1plq	247 / 1.380383 / 23.665969	248 / 1.37	B
1b77A - 1czdA	228 / 0.600158 / 26.084288	228 / 0.60	L
1b77A - 1dmlA	128 / 2.315777 / 4.719415	208 / 4.18	L
1b77A - 1ge8A	171 / 2.654882 / 8.227131	197 / 3.14	L
1b77A - 1plq	182 / 2.497389 / 9.368854	211 / 3.59	L
1czdA - 1dmlA	131 / 2.422025 / 4.543821	205 / 4.10	L
1czdA - 1ge8A	169 / 2.521506 / 8.428429	199 / 3.21	L
1czdA - 1plq	167 / 2.215476 / 8.830384	194 / 3.43	L
1dmlA - 1ge8A	158 / 2.745198 / 4.581019	220 / 4.06	L
1dmlA - 1plq	175 / 2.704235 / 5.639986	225 / 3.66	L
1ge8A - 1plq	221 / 1.455481 / 18.678872	237 / 1.95	L
**2.40.20**			
1b2iA - 1ceaA	78 / 1.601455 / 11.724877	79 / 1.60	L
1b2iA - 1kdu	68 / 2.102600 / 5.821307	73 / 2.64	L
1b2iA - 1kiv	74 / 1.611512 / 10.612841	76 / 1.69	L
1b2iA - 1krn	77 / 1.705257 / 10.486513	71 / 1.63	L
1b2iA - 1pk4	78 / 1.796284 / 11.695856	71 / 1.62	L
1b2iA - 1pmlA	75 / 1.959124 / 9.030821	81 / 2.36	L

1ceaA - 1kdu	70 / 2.070622 / 6.506356	76 / 2.39	L
1ceaA - 1kiv	77 / 0.905185 / 14.617575	75 / 0.84	L
1ceaA - 1krn	78 / 0.935444 / 14.164424	78 / 0.94	L
1ceaA - 1pk4	78 / 0.915780 / 15.184383	78 / 0.92	L
1ceaA - 1pmlA	73 / 1.441332 / 10.625511	79 / 1.80	L
1kdu - 1kiv	69 / 2.018335 / 6.654320	76 / 2.35	L
1kdu - 1krn	70 / 1.982801 / 6.045239	77 / 2.48	L
1kdu - 1pk4	69 / 2.016846 / 6.589132	77 / 2.47	L
1kdu - 1pmlA	73 / 1.858452 / 8.168420	80 / 2.08	L
1kiv - 1krn	78 / 0.637040 / 15.180550	78 / 0.64	L
1kiv - 1pk4	78 / 0.595011 / 16.300816	78 / 0.60	L
1kiv - 1pmlA	73 / 1.357903 / 11.411978	78 / 1.78	L
1krn - 1pk4	79 / 0.152775 / 17.384303	79 / 0.15	L
1krn - 1pmlA	75 / 1.520742 / 10.945835	79 / 1.85	L
1pk4 - 1pmlA	74 / 1.424190 / 11.591004	79 / 1.87	L

Comparison with SSM

Fisher's Dataset

Sequence Neighborhood based Program

PDBid1-PDBid2	Matchprot2 (Lali / RMSD / Zscore)	SSM (Lali / RMSD)	Good / Bad / Level
1dxtB - 1hbg	117 / 1.767981 / 10.883261	132 / 1.7492436033	B
1cpcL - 1colA	89 / 2.345877 / 2.543957	108 / 3.1063507269	L
1c2rA - 1ycc	94 / 1.824597 / 10.278438	93 / 1.3972064296	L
2mtaC - 1ycc	74 / 1.907191 / 4.444056	81 / 2.0764390788	L
1bbhA - 2ccyA	116 / 1.603838 / 13.049425	124 / 2.0408011967	L
1bgeB - 2gmfA	85 / 2.480859 / 3.803303	44 / 2.490543583	G
1rcb - 2gmfA	76 / 2.211365 / 3.873671	70 / 1.9819313071	L
1aep - 256bA	71 / 1.938330 / 3.276880	68 / 1.6560544163	L
1osa - 4cpv	66 / 1.274414 / 5.304187	65 / 1.3488192786	G
2sas - 2scpA	122 / 2.229376 / 7.007117	147 / 2.9526250685	L
1hom - 1lfb	54 / 1.522779 / 5.542545	53 / 1.3938619451	L
1lgaA - 2cyp	242 / 1.768907 / 15.013091	227 / 1.996795035	G
2hpdA - 2cpp	307 / 2.487783 / 8.767392	325 / 3.0085131148	L
1fc1A - 2fb4H	109 / 1.894847 / 4.978134	115 / 2.2763032541	L
2fbjL - 8fabB	180 / 1.810727 / 12.524636	184 / 1.8997761449	L
1cid - 2rhe	91 / 2.059754 / 5.504575	89 / 2.3259795464	G
1pfc - 3hlaB	80 / 2.259232 / 5.208956	79 / 2.2306259574	L
1ten - 3hhrB	84 / 1.580910 / 7.230915	73 / 2.0987799273	G
1tlk - 2rhe	87 / 1.580035 / 9.762585	85 / 1.7074716737	G
3cd4 - 2rhe	88 / 1.381726 / 7.526783	87 / 2.4048229176	G
3hlaB - 2rhe	70 / 2.269583 / 3.701798	78 / 3.0820948516	L
1aaj - 1paz	80 / 1.564230 / 8.505390	76 / 1.4994820095	L
2afnA - 1aozA	233 / 2.098379 / 6.903389	226 / 2.2254920693	G

2azaA - 1paz	72 / 2.463617 / 2.403247	79 / 2.4119815988	B
4sbvA - 2tbvA	155 / 1.482077 / 10.225784	153 / 1.5692437775	G
1bbt1 - 2plv1	147 / 2.356716 / 4.801429	155 / 2.2719596773	B
1sacA - 2ayh	127 / 2.592137 / 5.091943	130 / 2.9380986343	L
1ltsD - 1bovA	63 / 2.317266 / 3.863577	61 / 1.8728678156	L
1tie - 4fgf	105 / 2.209304 / 7.883892	114 / 2.8538818262	L
8i1b - 4fgf	110 / 1.915536 / 11.460574	110 / 1.8820845343	L
1arb - 5ptp	162 / 2.171203 / 10.283039	170 / 2.3637700118	L
2sga - 5ptp	132 / 1.971517 / 10.002339	141 / 2.2646436924	L
2snv - 5ptp	117 / 2.368816 / 5.778181	121 / 2.694106864	L
1mdc - 1ifc	127 / 2.512997 / 4.864672	128 / 1.834606812	B
1mup - 1rbp	121 / 2.120736 / 9.168372	131 / 2.3077372066	L
2sim - 1nsbA	262 / 2.727954 / 8.422257	271 / 2.8637060839	L
1cauB - 1cauA	153 / 2.057075 / 11.342371	155 / 1.8619061466	B
2omf - 2por	240 / 2.259293 / 9.478756	246 / 2.2845386349	L
1chrA - 2mnr	340 / 1.647549 / 20.115848	333 / 1.7537561761	G
2mnr - 4enl	234 / 2.363370 / 6.154323	246 / 2.7284563178	L
3rubL - 6xia	147 / 2.609368 / 0.911725	202 / 4.0454870767	L
1crl - 1ede	160 / 2.503302 / 2.546601	188 / 3.8125226941	L
1tahA - 1tca	172 / 2.020544 / 7.212261	182 / 2.2843579369	L
1aba - 1ego	69 / 1.886953 / 6.671629	72 / 3.1927561238	L
1dsbA - 2trxA	69 / 2.141120 / 2.644885	73 / 2.4861467805	L
1gp1A - 2trxA	89 / 2.308867 / 5.505891	92 / 2.2832127586	B
1atnA - 1atr	264 / 2.099867 / 10.675943	267 / 2.445299631	L
1hrhA - 1rnh	108 / 1.545015 / 11.875600	109 / 1.5597118391	L

3chy - 2fox	91 / 2.370168 / 5.082222	105 / 3.4027474668	L
2ak3A - 1gky	125 / 2.537671 / 5.006488	131 / 2.9875358993	L
1gky - 3adk	124 / 2.093791 / 6.759818	137 / 2.7437234747	L
2cmd - 6ldh	267 / 2.034366 / 15.927041	269 / 2.0307088328	B
1eaf - 4cla	144 / 2.284240 / 6.764282	169 / 2.3944272364	L
2gbp - 2liv	167 / 2.600682 / 3.336421	185 / 4.0243449267	L
1mioC - 2minB	304 / 2.547204 / 4.974903	375 / 3.0678270809	L
2pia - 1fnb	186 / 2.057759 / 8.453938	196 / 2.0057434455	B
1gal - 3cox	372 / 2.337642 / 9.443108	371 / 2.6920323718	G
1npx - 3grs	333 / 2.251019 / 9.270818	329 / 2.4712969265	G
1fxiA - 1ubq	54 / 2.183049 / 2.676437	60 / 2.8673043143	L
1cewI - 1molA	68 / 1.804976 / 5.625186	79 / 2.1202823958	L
1stfI - 1molA	77 / 1.870025 / 7.557261	85 / 2.1378527931	L
2pna - 1shaA	81 / 1.856416 / 7.029939	89 / 2.3550606955	L
2sarA - 9rnt	61 / 2.485668 / 2.509794	70 / 2.9458133231	L
1onc - 7rsa	90 / 2.201218 / 6.191860	93 / 1.3078506302	B
5fd1 - 2fxb	52 / 1.684135 / 3.302135	54 / 2.2062539675	L
2hhmA - 1fbpA	198 / 2.395615 / 8.102476	207 / 2.3141922536	B
1hip - 2hipA	66 / 1.716334 / 9.245717	62 / 1.4469591212	L
1isuA - 2hipA	54 / 1.692477 / 6.430629	- / -	G

Structure Neighborhood Based program

PDBid1 - PDBid2	Matchprot lali / rmsd / zscore	SSM (Lali / RMSD)	Good / Bad / Level
1dxtB - 1hbg	133 / 1.618052 / 15.527590	132 / 1.7492436033	G
1cpcL - 1colA	91 / 2.453736 / 2.658678	108 / 3.1063507269	L
1c2rA - 1ycc	95 / 1.362028 / 12.269984	93 / 1.3972064296	G
2mtaC - 1ycc	78 / 1.941872 / 4.895345	81 / 2.0764390788	L
1bbhA - 2ccyA	121 / 1.764434 / 13.536841	124 / 2.0408011967	L
1bgeB - 2gmfA	87 / 2.225433 / 5.446246	44 / 2.490543583	G
1rcb - 2gmfA	77 / 1.879182 / 5.082635	70 / 1.9819313071	G
1aep - 256bA	64 / 2.117783 / 1.639862	68 / 1.6560544163	B
1osa - 4cpv	65 / 1.526141 / 4.545486	65 / 1.3488192786	B
2sas - 2scpA	138 / 2.231157 / 9.750968	147 / 2.9526250685	L
1hom - 1lfb	52 / 1.281308 / 5.652445	53 / 1.3938619451	L
1lgaA - 2cyp	238 / 2.192340 / 12.818557	227 / 1.996795035	L
2hpdA - 2cpp	283 / 2.585693 / 6.934695	325 / 3.0085131148	L
1fc1A - 2fb4H	112 / 1.892321 / 4.922228	115 / 2.2763032541	L
2fbjL - 8fabB	185 / 1.735389 / 13.378943	184 / 1.8997761449	G
1cid - 2rhe	91 / 2.068938 / 5.552895	89 / 2.3259795464	G
1pfc - 3hlaB	85 / 2.230198 / 6.537908	79 / 2.2306259574	G
1ten - 3hhrB	82 / 1.392378 / 7.169996	73 / 2.0987799273	G
1tlk - 2rhe	84 / 1.347123 / 10.106946	85 / 1.7074716737	L
3cd4 - 2rhe	88 / 1.596862 / 6.705303	87 / 2.4048229176	G
3hlaB - 2rhe	68 / 2.266716 / 3.652379	78 / 3.0820948516	L
1aaj - 1paz	80 / 1.488212 / 8.657101	76 / 1.4994820095	G
2afnA - 1aozA	227 / 2.134964 / 5.970371	226 / 2.2254920693	G

2azaA - 1paz	79 / 2.208511 / 4.535960	79 / 2.4119815988	G
4sbvA - 2tbvA	154 / 1.573555 / 10.004015	153 / 1.5692437775	L
1bbt1 - 2plv1	156 / 2.045998 / 7.154092	155 / 2.2719596773	G
1sacA - 2ayh	139 / 2.453903 / 7.253736	130 / 2.9380986343	G
1ltsD - 1bovA	67 / 1.769267 / 6.901099	61 / 1.8728678156	G
1tie - 4fgf	106 / 2.279553 / 8.261742	114 / 2.8538818262	L
8i1b - 4fgf	110 / 1.901800 / 11.803427	110 / 1.8820845343	L
1arb - 5ptp	168 / 1.986419 / 11.874460	170 / 2.3637700118	L
2sga - 5ptp	129 / 1.833044 / 10.346103	141 / 2.2646436924	L
2snv - 5ptp	96 / 2.290828 / 3.292010	121 / 2.694106864	L
1mdc - 1ifc	- / - / -	128 / 1.834606812	B
1mup - 1rbp	122 / 2.009872 / 10.061081	131 / 2.3077372066	L
2sim - 1nsbA	262 / 2.632144 / 8.488201	271 / 2.8637060839	L
1cauB - 1cauA	152 / 1.738604 / 13.203211	155 / 1.8619061466	L
2omf - 2por	235 / 2.467897 / 8.573208	246 / 2.2845386349	B
1chrA - 2mnr	335 / 1.664985 / 19.321587	333 / 1.7537561761	L
2mnr - 4enl	230 / 2.517675 / 5.566740	246 / 2.7284563178	L
3rubL - 6xia	152 / 2.796273 / 0.795955	202 / 4.0454870767	L
1crl - 1ede	174 / 2.491762 / 3.063029	188 / 3.8125226941	L
1tahA - 1tca	168 / 2.416043 / 5.176979	182 / 2.2843579369	B
1aba - 1ego	69 / 2.000439 / 6.547013	72 / 3.1927561238	L
1dsbA - 2trxA	76 / 1.839218 / 4.229998	73 / 2.4861467805	G
1gp1A - 2trxA	86 / 1.886982 / 5.712329	92 / 2.2832127586	L
1atnA - 1atr	260 / 2.181434 / 10.128426	267 / 2.445299631	L
1hrhA - 1rnh	108 / 1.511972 / 12.136479	109 / 1.5597118391	L

3chy - 2fox	91 / 2.430114 / 5.063224	105 / 3.4027474668	L
2ak3A - 1gky	135 / 2.352513 / 6.312533	131 / 2.9875358993	G
1gky - 3adk	141 / 2.253797 / 8.316162	137 / 2.7437234747	G
2cmd - 6ldh	274 / 2.056520 / 16.639841	269 / 2.0307088328	G
1eaf - 4cla	160 / 2.022917 / 10.536119	169 / 2.3944272364	L
2gbp - 2liv	169 / 2.544403 / 4.131064	185 / 4.0243449267	L
1mioC - 2minB	318 / 2.330087 / 5.902166	375 / 3.0678270809	L
2pia - 1fnb	195 / 1.855691 / 9.533363	196 / 2.0057434455	L
1gal - 3cox	366 / 2.119449 / 10.096328	371 / 2.6920323718	L
1npx - 3grs	345 / 2.197732 / 10.535785	329 / 2.4712969265	G
1fxiA - 1ubq	56 / 2.163154 / 3.071374	60 / 2.8673043143	L
1cewI - 1molA	79 / 1.944987 / 7.305458	79 / 2.1202823958	G
1stfI - 1molA	82 / 1.852846 / 9.165097	85 / 2.1378527931	L
2pna - 1shaA	83 / 1.941611 / 7.092368	89 / 2.3550606955	L
2sarA - 9rnt	64 / 2.298871 / 3.773092	70 / 2.9458133231	L
1onc - 7rsa	93 / 1.296087 / 12.070139	93 / 1.3078506302	L
5fd1 - 2fxb	55 / 1.879364 / 3.600283	54 / 2.2062539675	G
2hhmA - 1fbpA	187 / 2.103918 / 8.559585	207 / 2.3141922536	L
1hip - 2hipA	65 / 1.462879 / 8.980166	62 / 1.4469591212	L
1isuA - 2hipA	53 / 1.497409 / 6.679889	- / -	G

Novotny's Dataset

Sequence Neighborhood based program

PDBid1 - PDBid2	Matchprot2 (Seq. Nbhd.) (Lali / RMSD / Zscore)	SSM (Lali / RMSD)	Good / Bad / Level
**1.10.164**			
1aq6A - 1c3uA	52 / 2.552922 / -1.412559	61 / 3.2456383813	L
1aq6A - 1fezA	161 / 1.993285 / 9.783233	177 / 2.9433658701	L
1aq6A - 1jud	217 / 1.304811 / 22.383812	217 / 1.279280364	B
1aq6A - 1zrn	217 / 1.292294 / 22.690917	215 / 1.2547787522	L
1c3uA - 1fezA	55 / 2.231143 / -1.211438	81 / 3.7146602919	L
1c3uA - 1jud	15 / 0.953262 / -1.856251	68 / 3.4835067793	L
1c3uA - 1zrn	78 / 2.630662 / -0.834005	71 / 3.6542215388	G
1fezA - 1jud	157 / 1.974788 / 9.996566	152 / 2.4639145732	G
1fezA - 1zrn	163 / 2.162118 / 10.277151	152 / 2.4639145732	G
1jud - 1zrn	220 / 0.265135 / 30.214916	220 / 0.2651345259	L
**1.10.40**			
1rlr - 1yfm	95 / 3.074015 / -1.563337	79 / 4.2704877504	G
1rlr - 1furA	85 / 2.771679 / -1.603553	68 / 3.5068431563	G
1rlr - 1auwA	85 / 2.734123 / -1.419069	75 / 4.5785495777	G
1rlr - 1jswA	86 / 2.680286 / -1.513539	95 / 4.5680614173	L
1rlr - 1hylA	62 / 2.972107 / -1.588022	90 / 5.4063409913	L
1rlr - 1i0aA	79 / 2.859903 / -1.749958	65 / 3.9160489988	G
1yfm - 1furA	446 / 1.007248 / 22.026687	446 / 1.0076740812	L
1yfm - 1auwA	347 / 2.131289 / 9.574798	323 / 2.3230784565	G
1yfm - 1jswA	423 / 1.807071 / 16.095213	430 / 1.9264287295	L
1yfm - 1hylA	55 / 2.680767 / -1.400364	56 / 5.5389453269	L
1yfm - 1i0aA	350 / 1.964233 / 10.218229	329 / 2.609571188	G

1furA - 1auwA	342 / 1.942800 / 10.100266	323 / 2.2554788215	G
1furA - 1jswA	413 / 1.749739 / 16.810692	429 / 2.3229816571	L
1furA - 1hylA	64 / 2.901541 / -1.251467	59 / 4.4252552159	G
1furA - 1i0aA	333 / 2.017358 / 9.289516	323 / 2.2219632467	G
1auwA - 1jswA	297 / 2.402125 / 6.362020	324 / 2.4920165768	L
1auwA - 1hylA	52 / 2.876209 / -1.581114	66 / 4.6262617184	L
1auwA - 1i0aA	445 / 0.681138 / 21.504745	434 / 0.56245343552	L
1jswA - 1hylA	59 / 2.819941 / -1.492591	65 / 5.717756208	L
1jswA - 1i0aA	314 / 2.260573 / 7.221459	322 / 2.4110755039	L
1hylA - 1i0aA	49 / 2.787425 / -1.673912	68 / 5.4405043543	L
**1.25.30**			
1b3uA - 1bk6A	197 / 2.649229 / 1.077622	186 / 5.5219310797	G
1b3uA - 1gcjA	249 / 2.731531 / 2.003215	324 / 4.3934408173	L
1b3uA - 1ialA	202 / 2.696890 / 1.073810	186 / 4.6225973403	G
1b3uA - 1ibrA	67 / 2.743584 / -0.973410	46 / 3.2551207782	G
1b3uA - 1qbkB	225 / 2.930389 / -0.880217	275 / 4.2358054843	L
1b3uA - 2bct	177 / 2.817308 / -0.072292	188 / 5.4042020421	L
1bk6A - 1gcjA	245 / 2.452586 / 3.966035	244 / 3.2988003133	G
1bk6A - 1ialA	396 / 1.654236 / 18.426181	401 / 1.7772160819	L
1bk6A - 1ibrA	59 / 2.883340 / -1.146076	49 / 4.2903031176	G
1bk6A - 1qbkB	235 / 2.739448 / 0.602050	259 / 3.4270396608	L
1bk6A - 2bct	288 / 2.368801 / 6.058353	377 / 3.2489758405	L
1gcjA - 1ialA	254 / 2.535090 / 4.109992	243 / 3.1280126384	G
1gcjA - 1ibrA	67 / 2.792291 / -1.024028	40 / 2.6111111496	L
1gcjA - 1qbkB	310 / 2.385825 / 2.201474	267 / 3.458591862	G

1gcjA - 2bct	226 / 2.833470 / 1.579869	234 / 3.4111431653	L
1ialA - 1ibrA	46 / 2.691598 / -1.501617	54 / 3.7335661835	L
1ialA - 1qbkB	227 / 2.665372 / 0.732473	295 / 4.0911502048	L
1ialA - 2bct	269 / 2.223326 / 5.451147	357 / 3.3305799805	L
1ibrA - 1qbkB	66 / 2.750472 / -1.307502	60 / 3.5519181663	G
1ibrA - 2bct	69 / 2.781739 / -0.963062	48 / 3.4529143874	G
1qbkB - 2bct	231 / 2.661437 / 0.222369	281 / 4.1088276971	L
**2.30.110**			
1ci0A - 1dnlA	191 / 1.440915 / 18.765724	193 / 1.4067266688	B
1ci0A - 1ejeA	90 / 1.893767 / 4.037346	87 / 2.070111088	G
1ci0A - 1i0rA	78 / 1.867700 / 3.360269	85 / 2.0913202868	L
1dnlA - 1ejeA	82 / 1.700216 / 3.516565	86 / 1.9377129267	L
1dnlA - 1i0rA	75 / 1.716565 / 2.999491	88 / 2.2688841933	L
1ejeA - 1i0rA	139 / 1.617463 / 14.237837	144 / 1.8479964857	L
**2.40.100**			
1a33 - 1cynA	165 / 0.831118 / 25.581584	163 / 0.75440118433	L
1a33 - 1dywA	167 / 0.766534 / 27.063242	168 / 0.80537325903	L
1a33 - 1ihgA	173 / 0.764321 / 17.459092	172 / 0.7146497071	L
1a33 - 1lopA	140 / 1.139730 / 17.874507	138 / 1.1011088161	L
1a33 - 1qngA	166 / 0.917395 / 26.507498	165 / 0.85656775883	L
1a33 - 1qoiA	168 / 1.049905 / 25.745149	167 / 0.97166960767	L
1a33 - 2rmcA	165 / 0.811267 / 25.329352	163 / 0.7276266867	L
1cynA - 1dywA	163 / 0.873024 / 25.377609	160 / 0.6378450867	L
1cynA - 1ihgA	167 / 0.812465 / 16.103729	164 / 0.65015257252	L
1cynA - 1lopA	144 / 1.264397 / 18.516408	138 / 0.98428896529	L

1cynA - 1qngA	162 / 0.807030 / 25.441336	159 / 0.61862084915	L
1cynA - 1qoiA	164 / 0.711896 / 25.489766	163 / 0.71760627645	L
1cynA - 2rmcA	176 / 0.489924 / 28.988623	175 / 0.4501550688	L
1dywA - 1ihgA	170 / 0.784628 / 17.359629	168 / 0.6206927432	L
1dywA - 1lopA	141 / 1.354386 / 17.440849	134 / 1.1159342016	L
1dywA - 1qngA	168 / 0.640550 / 28.546519	168 / 0.6319617028	L
1dywA - 1qoiA	164 / 1.033299 / 25.202564	162 / 0.90775889032	L
1dywA - 2rmcA	159 / 0.634684 / 24.415682	160 / 0.71399466606	L
1ihgA - 1lopA	143 / 1.271632 / 11.138583	139 / 1.2099423031	L
1ihgA - 1qngA	167 / 0.840740 / 16.883894	165 / 0.67203149632	L
1ihgA - 1qoiA	165 / 0.755089 / 16.185676	164 / 0.72858452112	L
1ihgA - 2rmcA	166 / 0.842582 / 15.402875	164 / 0.75103735284	L
1lopA - 1qngA	141 / 1.528718 / 17.161924	132 / 1.0927971587	L
1lopA - 1qoiA	140 / 1.102437 / 18.060041	137 / 1.1252237337	G
1lopA - 2rmcA	143 / 1.216579 / 17.977920	139 / 1.1591623311	L
1qngA - 1qoiA	166 / 0.931126 / 26.442762	165 / 0.89751503734	L
1qngA - 2rmcA	162 / 0.929837 / 24.657075	159 / 0.72428419465	L
1qoiA - 2rmcA	165 / 0.870222 / 24.725030	162 / 0.76096771092	L
**2.100.10**			
1c3kA - 1ciy	118 / 2.517169 / 2.564285	124 / 2.3506661555	B
1c3kA - 1jacA	125 / 1.447366 / 15.925442	124 / 1.4951384631	G
1c3kA - 1jotA	127 / 1.528057 / 16.300306	124 / 1.4740816425	L
1c3kA - 1dlc	125 / 2.492074 / 2.989941	134 / 2.5020022465	L
1c3kA - 1vmoA	120 / 2.312039 / 9.734756	130 / 2.8442338067	L
1ciy - 1jacA	109 / 2.464506 / 2.133106	107 / 1.9973795144	L

1ciy - 1jotA	109 / 2.529496 / 2.042840	112 / 2.1598342685	B
1ciy - 1dlc	539 / 1.589934 / 17.736200	535 / 1.5745264427	L
1ciy - 1vmoA	125 / 2.589238 / 2.103541	128 / 3.6470933451	L
1jacA - 1jotA	133 / 0.360512 / 24.617682	133 / 0.36051222794	L
1jacA - 1dlc	115 / 2.684532 / 1.764475	119 / 2.5017579025	B
1jacA - 1vmoA	105 / 2.472352 / 6.645189	118 / 2.899112652	L
1jotA - 1dlc	112 / 2.607978 / 1.779138	119 / 2.4862281762	B
1jotA - 1vmoA	101 / 2.366930 / 6.039785	116 / 2.8858592881	L
1dlc - 1vmoA	121 / 2.811144 / 1.283490	115 / 3.3887768689	L
**3.10.70**			
1bkf - 1grj	53 / 2.129308 / 1.518653	53 / 2.4070023334	G
1bkf - 1pbk	106 / 1.031962 / 17.559391	105 / 1.0147585928	L
1bkf - 1rot	105 / 1.586925 / 14.708936	103 / 1.4939336839	L
1bkf - 1yat	107 / 0.740635 / 18.854510	106 / 0.73398781516	L
1grj - 1pbk	48 / 2.230752 / 0.214513	53 / 2.6642989915	L
1grj - 1rot	49 / 2.533945 / -0.305146	56 / 2.8625339355	L
1grj - 1yat	53 / 2.097045 / 1.407873	55 / 2.6778292266	L
1pbk - 1rot	103 / 1.447314 / 13.590910	103 / 1.5722028373	G
1pbk - 1yat	107 / 0.956091 / 17.711989	106 / 0.95334194466	L
1rot - 1yat	111 / 1.526901 / 15.852777	110 / 1.480187703	L
**3.40.91**			
1bhmA - 1cfr	92 / 2.541242 / 1.558147	97 / 3.2338449544	L
1bhmA - 1d2iA	146 / 1.851088 / 10.514207	137 / 1.92596925	G
1bhmA - 1fokA	84 / 2.652422 / -0.426451	98 / 3.4190929754	L
1cfr - 1d2iA	88 / 2.182160 / 1.681056	97 / 3.0928248144	L

1cfr - 1fokA	106 / 2.600421 / 0.036905	114 / 3.5254913136	L
1d2iA - 1fokA	91 / 2.755003 / -0.468240	91 / 3.1582248715	G
**3.70.10**			
1axcA - 1b77A	168 / 2.429400 / 8.210375	191 / 3.019710106	L
1axcA - 1czdA	174 / 2.324310 / 9.465431	191 / 2.8700362099	L
1axcA - 1dmlA	181 / 2.777907 / 6.577876	210 / 3.4309514726	L
1axcA - 1ge8A	229 / 1.471087 / 21.067522	224 / 1.4552782741	L
1axcA - 1plq	247 / 1.327445 / 23.877194	247 / 1.3329808215	L
1b77A - 1czdA	228 / 0.600158 / 26.084288	228 / 0.60015884812	L
1b77A - 1dmlA	147 / 2.871160 / 3.251943	182 / 3.9246171209	L
1b77A - 1ge8A	169 / 2.436944 / 8.313997	185 / 2.7406090291	L
1b77A - 1plq	173 / 2.353772 / 9.372677	187 / 2.6966659958	L
1czdA - 1dmlA	152 / 2.828240 / 3.726109	181 / 3.914512248	L
1czdA - 1ge8A	178 / 2.466398 / 9.430839	185 / 2.6765183039	L
1czdA - 1plq	177 / 2.344821 / 9.793609	188 / 2.7410168547	L
1dmlA - 1ge8A	165 / 2.723948 / 4.926716	207 / 3.6619087013	L
1dmlA - 1plq	177 / 2.827262 / 6.036022	209 / 3.4895576921	L
1ge8A - 1plq	222 / 1.595009 / 18.242090	223 / 1.4466669178	B
**2.40.20**			
1b2iA - 1ceaA	71 / 1.624921 / 9.262427	78 / 1.556492639	B
1b2iA - 1kdu	68 / 2.131224 / 5.320875	72 / 2.5399075295	L
1b2iA - 1kiv	76 / 1.632192 / 11.183288	78 / 1.7708094831	L
1b2iA - 1krn	77 / 1.721116 / 10.402196	78 / 1.8286517512	L
1b2iA - 1pk4	75 / 1.711139 / 10.876151	78 / 1.815802268	L
1b2iA - 1pmlA	75 / 1.991277 / 8.618455	75 / 1.9182850403	B

1ceaA - 1kdu	69 / 2.235091 / 5.622503	69 / 2.021506592	B
1ceaA - 1kiv	77 / 0.924612 / 14.632626	78 / 0.98145690519	L
1ceaA - 1krn	78 / 0.935780 / 14.157968	79 / 1.0067834027	L
1ceaA - 1pk4	78 / 0.915780 / 15.184383	79 / 0.98002584223	L
1ceaA - 1pmlA	74 / 1.504394 / 10.624290	75 / 1.43215263	B
1kdu - 1kiv	71 / 2.130127 / 6.895939	69 / 2.053218388	L
1kdu - 1krn	63 / 1.793541 / 4.976156	68 / 1.9967368282	L
1kdu - 1pk4	68 / 2.072544 / 6.073593	68 / 2.038604836	L
1kdu - 1pmlA	72 / 1.815999 / 8.075270	77 / 1.9490929659	L
1kiv - 1krn	78 / 0.637152 / 15.177455	78 / 0.63728304469	L
1kiv - 1pk4	78 / 0.595011 / 16.300816	78 / 0.59501347616	L
1kiv - 1pmlA	73 / 1.357903 / 11.411978	75 / 1.5220734164	L
1krn - 1pk4	79 / 0.152950 / 17.384359	79 / 0.15395647511	L
1krn - 1pmlA	73 / 1.826699 / 8.854390	73 / 1.3950516456	B
1pk4 - 1pmlA	73 / 1.425936 / 11.459319	73 / 1.361892868	B

Structure Neighborhood based program

PDBid1 - PDBid2	Matchprot2 (Struct. Nbhd.) (Lali / RMSD / Zscore)	SSM (Lali / RMSD)	Good / Bad / Level
**1.10.164**			
1aq6A - 1c3uA	66 / 2.457820 / -0.975812	61 / 3.2456383813	G
1aq6A - 1fezA	168 / 2.175743 / 10.014351	177 / 2.9433658701	L
1aq6A - 1jud	216 / 1.232942 / 22.438126	217 / 1.279280364	L
1aq6A - 1zrn	216 / 1.232157 / 22.631065	215 / 1.2547787522	G
1c3uA - 1fezA	76 / 2.204634 / -0.478927	81 / 3.7146602919	L
1c3uA - 1jud	7 / 0.195000 / -1.954991	68 / 3.4835067793	L
1c3uA - 1zrn	8 / 0.285602 / -1.941053	71 / 3.6542215388	L
1fezA - 1jud	157 / 2.020545 / 9.813373	152 / 2.4639145732	G
1fezA - 1zrn	159 / 2.043136 / 10.014177	152 / 2.4639145732	G
1jud - 1zrn	220 / 0.265135 / 30.214916	220 / 0.2651345259	L
**1.10.40**			
1rlr - 1yfm	79 / 2.457731 / -1.531181	79 / 4.2704877504	G
1rlr - 1furA	83 / 2.263151 / -1.504402	68 / 3.5068431563	G
1rlr - 1auwA	88 / 2.666284 / -1.397462	75 / 4.5785495777	G
1rlr - 1jswA	81 / 2.525280 / -1.547428	95 / 4.5680614173	L
1rlr - 1hylA	11 / 1.016705 / -1.940894	90 / 5.4063409913	L
1rlr - 1i0aA	77 / 2.455953 / -1.621795	65 / 3.9160489988	G
1yfm - 1furA	446 / 1.007248 / 22.026687	446 / 1.0076740812	L
1yfm - 1auwA	344 / 1.961941 / 10.112582	323 / 2.3230784565	G
1yfm - 1jswA	423 / 1.738966 / 16.686973	430 / 1.9264287295	L
1yfm - 1hylA	7 / 0.704219 / -1.965680	56 / 5.5389453269	L
1yfm - 1i0aA	344 / 2.157840 / 9.397772	329 / 2.609571188	G

1furA - 1auwA	338 / 2.018078 / 9.832389	323 / 2.2554788215	G
1furA - 1jswA	413 / 1.812258 / 16.035052	429 / 2.3229816571	L
1furA - 1hylA	11 / 0.743377 / -1.918575	59 / 4.4252552159	L
1furA - 1i0aA	350 / 2.094681 / 10.328766	323 / 2.2219632467	G
1auwA - 1jswA	322 / 2.182510 / 7.833997	324 / 2.4920165768	L
1auwA - 1hylA	7 / 0.550223 / -1.962580	66 / 4.6262617184	L
1auwA - 1i0aA	445 / 0.660635 / 21.532919	434 / 0.56245343552	L
1jswA - 1hylA	18 / 2.069607 / -1.892759	65 / 5.717756208	L
1jswA - 1i0aA	330 / 2.235845 / 8.207263	322 / 2.4110755039	L
1hylA - 1i0aA	13 / 1.311338 / -1.891736	68 / 5.4405043543	L
**1.25.30**			
1b3uA - 1bk6A	203 / 2.759172 / 1.192922	186 / 5.5219310797	L
1b3uA - 1gcjA	223 / 2.573049 / 1.412590	324 / 4.3934408173	L
1b3uA - 1ialA	195 / 2.788175 / 0.715012	186 / 4.6225973403	G
1b3uA - 1ibrA	16 / 1.197663 / -1.869836	46 / 3.2551207782	L
1b3uA - 1qbkB	202 / 2.707436 / -0.849227	275 / 4.2358054843	L
1b3uA - 2bct	176 / 2.616014 / 0.237968	188 / 5.4042020421	L
1bk6A - 1gcjA	249 / 2.446925 / 4.374222	244 / 3.2988003133	G
1bk6A - 1ialA	394 / 1.629739 / 18.051942	401 / 1.7772160819	L
1bk6A - 1ibrA	17 / 1.657662 / -1.794449	49 / 4.2903031176	L
1bk6A - 1qbkB	244 / 2.571325 / 1.146742	259 / 3.4270396608	L
1bk6A - 2bct	308 / 2.553301 / 6.277557	377 / 3.2489758405	L
1gcjA - 1ialA	244 / 2.565092 / 3.769208	243 / 3.1280126384	G
1gcjA - 1ibrA	43 / 2.214131 / -1.274271	40 / 2.6111111496	L
1gcjA - 1qbkB	260 / 2.651475 / 1.230772	267 / 3.458591862	L

1gcjA - 2bct	231 / 2.426738 / 2.757577	234 / 3.4111431653	L
1ialA - 1ibrA	15 / 0.825930 / -1.798085	54 / 3.7335661835	L
1ialA - 1qbkB	254 / 2.724410 / 0.985744	295 / 4.0911502048	L
1ialA - 2bct	286 / 2.342282 / 5.748118	357 / 3.3305799805	L
1ibrA - 1qbkB	33 / 1.910666 / -1.685608	60 / 3.5519181663	L
1ibrA - 2bct	8 / 0.373141 / -1.940882	48 / 3.4529143874	L
1qbkB - 2bct	224 / 2.603785 / 0.147578	281 / 4.1088276971	L
**2.30.110**			
1ci0A - 1dnlA	191 / 1.465403 / 18.749262	193 / 1.4067266688	B
1ci0A - 1ejeA	83 / 1.754242 / 3.534641	87 / 2.070111088	L
1ci0A - 1i0rA	83 / 1.909922 / 3.818560	85 / 2.0913202868	L
1dnlA - 1ejeA	81 / 1.618309 / 3.399219	86 / 1.9377129267	L
1dnlA - 1i0rA	76 / 1.686808 / 2.943237	88 / 2.2688841933	L
1ejeA - 1i0rA	144 / 1.667200 / 14.952628	144 / 1.8479964857	G
**2.40.100**			
1a33 - 1cynA	164 / 0.729849 / 25.560363	163 / 0.75440118433	G
1a33 - 1dywA	170 / 0.939079 / 27.346710	168 / 0.80537325903	L
1a33 - 1ihgA	173 / 0.764321 / 17.459092	172 / 0.7146497071	L
1a33 - 1lopA	142 / 1.153245 / 18.416710	138 / 1.1011088161	L
1a33 - 1qngA	167 / 0.997673 / 26.510461	165 / 0.85656775883	L
1a33 - 1qoiA	167 / 1.169084 / 24.947909	167 / 0.97166960767	B
1a33 - 2rmcA	165 / 0.778459 / 25.405342	163 / 0.7276266867	L
1cynA - 1dywA	161 / 0.728774 / 25.253658	160 / 0.6378450867	L
1cynA - 1ihgA	167 / 0.807851 / 16.038324	164 / 0.65015257252	L
1cynA - 1lopA	143 / 1.204773 / 18.310080	138 / 0.98428896529	L

1cynA - 1qngA	163 / 0.878122 / 25.478743	159 / 0.61862084915	L
1cynA - 1qoiA	165 / 0.765248 / 25.616371	163 / 0.71760627645	L
1cynA - 2rmcA	176 / 0.489924 / 28.988623	175 / 0.4501550688	L
1dywA - 1ihgA	169 / 0.681296 / 17.367192	168 / 0.6206927432	L
1dywA - 1lopA	141 / 1.371839 / 17.660921	134 / 1.1159342016	L
1dywA - 1qngA	169 / 0.707653 / 28.690540	168 / 0.6319617028	L
1dywA - 1qoiA	164 / 1.019824 / 25.218047	162 / 0.90775889032	L
1dywA - 2rmcA	161 / 0.784063 / 24.592025	160 / 0.71399466606	L
1ihgA - 1lopA	142 / 1.200484 / 11.116520	139 / 1.2099423031	G
1ihgA - 1qngA	167 / 0.826290 / 16.990349	165 / 0.67203149632	L
1ihgA - 1qoiA	165 / 0.755089 / 16.185676	164 / 0.72858452112	L
1ihgA - 2rmcA	166 / 0.832548 / 15.404889	164 / 0.75103735284	L
1lopA - 1qngA	139 / 1.283402 / 17.698926	132 / 1.0927971587	L
1lopA - 1qoiA	142 / 1.236044 / 18.249012	137 / 1.1252237337	L
1lopA - 2rmcA	143 / 1.175040 / 18.156528	139 / 1.1591623311	L
1qngA - 1qoiA	166 / 0.934620 / 26.360490	165 / 0.89751503734	L
1qngA - 2rmcA	163 / 0.995364 / 24.698286	159 / 0.72428419465	L
1qoiA - 2rmcA	165 / 0.926091 / 24.565419	162 / 0.76096771092	L
**2.100.10**			
1c3kA - 1ciy	120 / 2.110024 / 3.368612	124 / 2.3506661555	L
1c3kA - 1jacA	125 / 1.435965 / 16.151930	124 / 1.4951384631	G
1c3kA - 1jotA	127 / 1.523956 / 16.300536	124 / 1.4740816425	L
1c3kA - 1dlc	124 / 2.337422 / 3.054529	134 / 2.5020022465	L
1c3kA - 1vmoA	126 / 2.564556 / 9.449866	130 / 2.8442338067	L
1ciy - 1jacA	111 / 2.090545 / 2.985861	107 / 1.9973795144	L

1ciy - 1jotA	111 / 2.210455 / 2.679259	112 / 2.1598342685	B
1ciy - 1dlc	541 / 1.566309 / 17.752113	535 / 1.5745264427	G
1ciy - 1vmoA	122 / 2.623253 / 2.103426	128 / 3.6470933451	L
1jacA - 1jotA	133 / 0.360512 / 24.617682	133 / 0.36051222794	L
1jacA - 1dlc	113 / 2.107684 / 3.212410	119 / 2.5017579025	L
1jacA - 1vmoA	100 / 2.325070 / 5.552021	118 / 2.899112652	L
1jotA - 1dlc	116 / 2.186783 / 3.209516	119 / 2.4862281762	L
1jotA - 1vmoA	110 / 2.533567 / 8.273235	116 / 2.8858592881	L
1dlc - 1vmoA	129 / 2.618661 / 2.558328	115 / 3.3887768689	G
**3.10.70**			
1bkf - 1grj	53 / 1.567967 / 2.578350	53 / 2.4070023334	G
1bkf - 1pbk	106 / 1.031962 / 17.559391	105 / 1.0147585928	L
1bkf - 1rot	104 / 1.471554 / 14.743686	103 / 1.4939336839	L
1bkf - 1yat	107 / 0.740635 / 18.854510	106 / 0.73398781516	L
1grj - 1pbk	51 / 1.281313 / 2.236802	53 / 2.6642989915	L
1grj - 1rot	47 / 1.641380 / 0.936361	56 / 2.8625339355	L
1grj - 1yat	53 / 1.512229 / 2.352950	55 / 2.6778292266	L
1pbk - 1rot	103 / 1.382043 / 13.859318	103 / 1.5722028373	G
1pbk - 1yat	107 / 0.957710 / 17.727875	106 / 0.95334194466	G
1rot - 1yat	109 / 1.433523 / 15.401467	110 / 1.480187703	L
**3.40.91**			
1bhmA - 1cfr	91 / 2.177180 / 2.025581	97 / 3.2338449544	L
1bhmA - 1d2iA	148 / 1.878168 / 10.815694	137 / 1.92596925	G
1bhmA - 1fokA	63 / 2.727453 / -1.303968	98 / 3.4190929754	L
1cfr - 1d2iA	92 / 2.400749 / 1.391207	97 / 3.0928248144	L

1cfr - 1fokA	113 / 2.541903 / 0.407745	114 / 3.5254913136	L
1d2iA - 1fokA	80 / 2.488961 / -0.469540	91 / 3.1582248715	L
**3.70.10**			
1axcA - 1b77A	174 / 2.451725 / 8.847162	191 / 3.019710106	L
1axcA - 1czdA	164 / 2.361052 / 8.345812	191 / 2.8700362099	L
1axcA - 1dmlA	183 / 2.760560 / 6.466431	210 / 3.4309514726	L
1axcA - 1ge8A	230 / 1.569787 / 21.042094	224 / 1.4552782741	L
1axcA - 1plq	247 / 1.380383 / 23.665969	247 / 1.3329808215	B
1b77A - 1czdA	228 / 0.600158 / 26.084288	228 / 0.60015884812	L
1b77A - 1dmlA	128 / 2.315777 / 4.719415	182 / 3.9246171209	L
1b77A - 1ge8A	171 / 2.654882 / 8.227131	185 / 2.7406090291	L
1b77A - 1plq	182 / 2.497389 / 9.368854	187 / 2.6966659958	G
1czdA - 1dmlA	131 / 2.422025 / 4.543821	181 / 3.914512248	L
1czdA - 1ge8A	169 / 2.521506 / 8.428429	185 / 2.6765183039	L
1czdA - 1plq	167 / 2.215476 / 8.830384	188 / 2.7410168547	L
1dmlA - 1ge8A	158 / 2.745198 / 4.581019	207 / 3.6619087013	L
1dmlA - 1plq	175 / 2.704235 / 5.639986	209 / 3.4895576921	L
1ge8A - 1plq	221 / 1.455481 / 18.678872	223 / 1.4466669178	B
**2.40.20**			
1b2iA - 1ceaA	78 / 1.601455 / 11.724877	78 / 1.556492639	B
1b2iA - 1kdu	68 / 2.102600 / 5.821307	72 / 2.5399075295	L
1b2iA - 1kiv	74 / 1.611512 / 10.612841	78 / 1.7708094831	L
1b2iA - 1krn	77 / 1.705257 / 10.486513	78 / 1.8286517512	L
1b2iA - 1pk4	78 / 1.796284 / 11.695856	78 / 1.815802268	G
1b2iA - 1pmlA	75 / 1.959124 / 9.030821	75 / 1.9182850403	L

1ceaA - 1kdu	70 / 2.070622 / 6.506356	69 / 2.021506592	L
1ceaA - 1kiv	77 / 0.905185 / 14.617575	78 / 0.98145690519	L
1ceaA - 1krn	78 / 0.935444 / 14.164424	79 / 1.0067834027	L
1ceaA - 1pk4	78 / 0.915780 / 15.184383	79 / 0.98002584223	L
1ceaA - 1pmlA	73 / 1.441332 / 10.625511	75 / 1.43215263	B
1kdu - 1kiv	69 / 2.018335 / 6.654320	69 / 2.053218388	L
1kdu - 1krn	70 / 1.982801 / 6.045239	68 / 1.9967368282	G
1kdu - 1pk4	69 / 2.016846 / 6.589132	68 / 2.038604836	G
1kdu - 1pmlA	73 / 1.858452 / 8.168420	77 / 1.9490929659	L
1kiv - 1krn	78 / 0.637040 / 15.180550	78 / 0.63728304469	L
1kiv - 1pk4	78 / 0.595011 / 16.300816	78 / 0.59501347616	L
1kiv - 1pmlA	73 / 1.357903 / 11.411978	75 / 1.5220734164	L
1krn - 1pk4	79 / 0.152775 / 17.384303	79 / 0.15395647511	L
1krn - 1pmlA	75 / 1.520742 / 10.945835	73 / 1.3950516456	L
1pk4 - 1pmlA	74 / 1.424190 / 11.591004	73 / 1.361892868	L
